# A versatile laser-induced porcine model of outer retinal and choroidal degeneration for preclinical testing

**DOI:** 10.1172/jci.insight.157654

**Published:** 2023-06-08

**Authors:** Francesca Barone, Juan Amaral, Irina Bunea, Mitra Farnoodian, Rohan Gupta, Rishabh Gupta, Dara Baker, M. Joseph Phillips, Richard J. Blanch, Arvydas Maminishkis, David M. Gamm, Kapil Bharti

**Affiliations:** 1 National Eye Institute (NEI), NIH, Bethesda, Maryland, USA.; 2 McPherson Eye Research Institute and Waisman Center, and; 3 Department of Ophthalmology and Visual Sciences, University of Wisconsin, Madison, Wisconsin, USA.; 4 Academic Department of Military Surgery and Trauma, Royal Centre for Defense Medicine, Birmingham, United Kingdom.; 5 Neuroscience and Ophthalmology, Institute of Inflammation and Ageing, University of Birmingham, Birmingham, United Kingdom.; 6 University Hospitals Birmingham NHS Foundation Trust, Birmingham, United Kingdom.

**Keywords:** Ophthalmology, Stem cells, Gene therapy, Retinopathy, Stem cell transplantation

## Abstract

Over 30 million people worldwide suffer from untreatable vision loss and blindness associated with childhood-onset and age-related eye diseases caused by photoreceptor (PR), retinal pigment epithelium (RPE), and choriocapillaris (CC) degeneration. Recent work suggests that RPE-based cell therapy may slow down vision loss in late stages of age-related macular degeneration (AMD), a polygenic disease induced by RPE atrophy. However, accelerated development of effective cell therapies is hampered by the lack of large-animal models that allow testing safety and efficacy of clinical doses covering the human macula (20 mm^2^). We developed a versatile pig model to mimic different types and stages of retinal degeneration. Using an adjustable power micropulse laser, we generated varying degrees of RPE, PR, and CC damage and confirmed the damage by longitudinal analysis of clinically relevant outcomes, including analyses by adaptive optics and optical coherence tomography/angiography, along with automated image analysis. By imparting a tunable yet targeted damage to the porcine CC and visual streak — with a structure similar to the human macula — this model is optimal for testing cell and gene therapies for outer retinal diseases including AMD, retinitis pigmentosa, Stargardt, and choroideremia. The amenability of this model to clinically relevant imaging outcomes will facilitate faster translation to patients.

## Graphical abstract

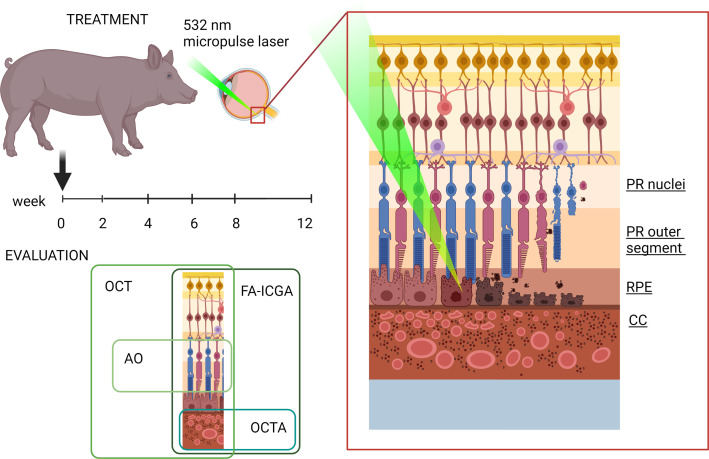

## Introduction

Retinal degenerative diseases, such as age-related macular degeneration (AMD), retinitis pigmentosa, Stargardt macular dystrophy, and choroideremia, are major causes of morbidity because visual impairment significantly decreases quality of life (1–4). Despite genetic and symptomatic diversity, photoreceptor (PR), retinal pigment epithelium (RPE), and choriocapillaris (CC) degeneration are common features of most outer retinal degenerations (5). Furthermore, outer retinal damage in battlefield and civilian ocular injuries is the most common cause of acute vision loss in younger populations (6). In the battlefield, these injuries occur through concussive or laser-based injuries (7). Cell and gene therapies and retinal prosthetics are promising therapeutic approaches currently being developed to restore vision in patients suffering from outer retinal damage or degeneration (8, 9). However, testing of the clinical dose of a transplant that, in some cases, can be as large as 20 mm^2^ (size of the human macula) is not feasible in small-animal models like rodents and rabbits. Furthermore, these small-animal models are not predictive of success in a human trial because of significant anatomical differences with the human eye (10, 11). Animal models of chemical injury — for instance, induced by sodium iodate and iodoacetic acid injections — also damage the inner retinal circuitry, making them less useful for outer retinal degenerations (12, 13). The lack of large-animal models of selective outer retinal degeneration to assess the safety and efficacy of cell therapies limits the therapeutic progress for outer retinal degenerative diseases.

The porcine eye presents an excellent preclinical model because: (a) the size and anatomical similarities to the human eye, including a central cone-rich retinal area free of large vessels (the visual streak) that resembles the human macula (14, 15); (b) it has an axial length of 23.9 ± 0.08 mm (mean ± SD), similar to the human eye, which is 23.67 ± 0.9 mm (16, 17); (c) it has a holangiotic retinal vascular system, similar to the human eye (18); (d) it lacks a tapetum, a noncellular tissue present underneath the RPE of other large animal eyes such as dogs and cats (19); (e) it has a suitability for vitrectomy and other posterior segment surgical manipulations allowing easier testing of therapeutic strategies; and (f) it is cost effective as compared with nonhuman primates (NHPs), both direct and indirect costs of research using NHP average 10***×*** higher compared with research with pigs. Overall, pig eye is an excellent model for testing treatment options being developed for humans.

A major challenge for performing preclinical research using the pig eye is the limited availability of genetic models with outer retinal degeneration. Transgenic pigs have been developed as models for some forms of retinitis pigmentosa due to RHODOPSIN (RHO) mutations (Pro23His, Pro347Ser112, and Pro347Leu mutations) (20, 21) and Stargardt disease caused by ELOVL4 mutations (22). However, transgenic pig work is limited due to the high cost, small litter numbers, long gestation, and long life span (23). Furthermore, transgenesis in pigs has a success rate between 0.6% and 8% (21, 23, 24). A clear demand for large-animal models to test human-specific clinical products and the advantages of pigs over NHPs indicates the need to develop a porcine model that can accommodate a wide variety of outer retinal treatment modalities.

Laser light at 532 nm is selectively absorbed by RPE pigment, heating RPE cells and causing them to undergo apoptosis (25–27). Recently, we investigated the therapeutic effect of induced pluripotent stem cell–derived RPE (iPSC-RPE) as a replacement tissue in a pig model of laser-induced RPE degeneration (27). This work highlighted the possibility of using a laser injury with varied laser intensity to develop a tunable outer retinal degeneration model such that damage could be targeted only to the RPE plus PR, and RPE plus PR plus CC. Use of clinically relevant imaging modalities, optical coherence tomography (OCT), OCT angiography (OCTA), fluoresceine angiography (FA), indocyanine green angiography (ICGA), and adaptive optics (AO) support rapid clinical translation to patients. We report the generation of a cost-effective pig model for testing clinical doses of retinal prosthetics as well as gene and cell replacement therapies.

## Results

### Micropulse laser–mediated outer retina injury.

**Figure 1:**
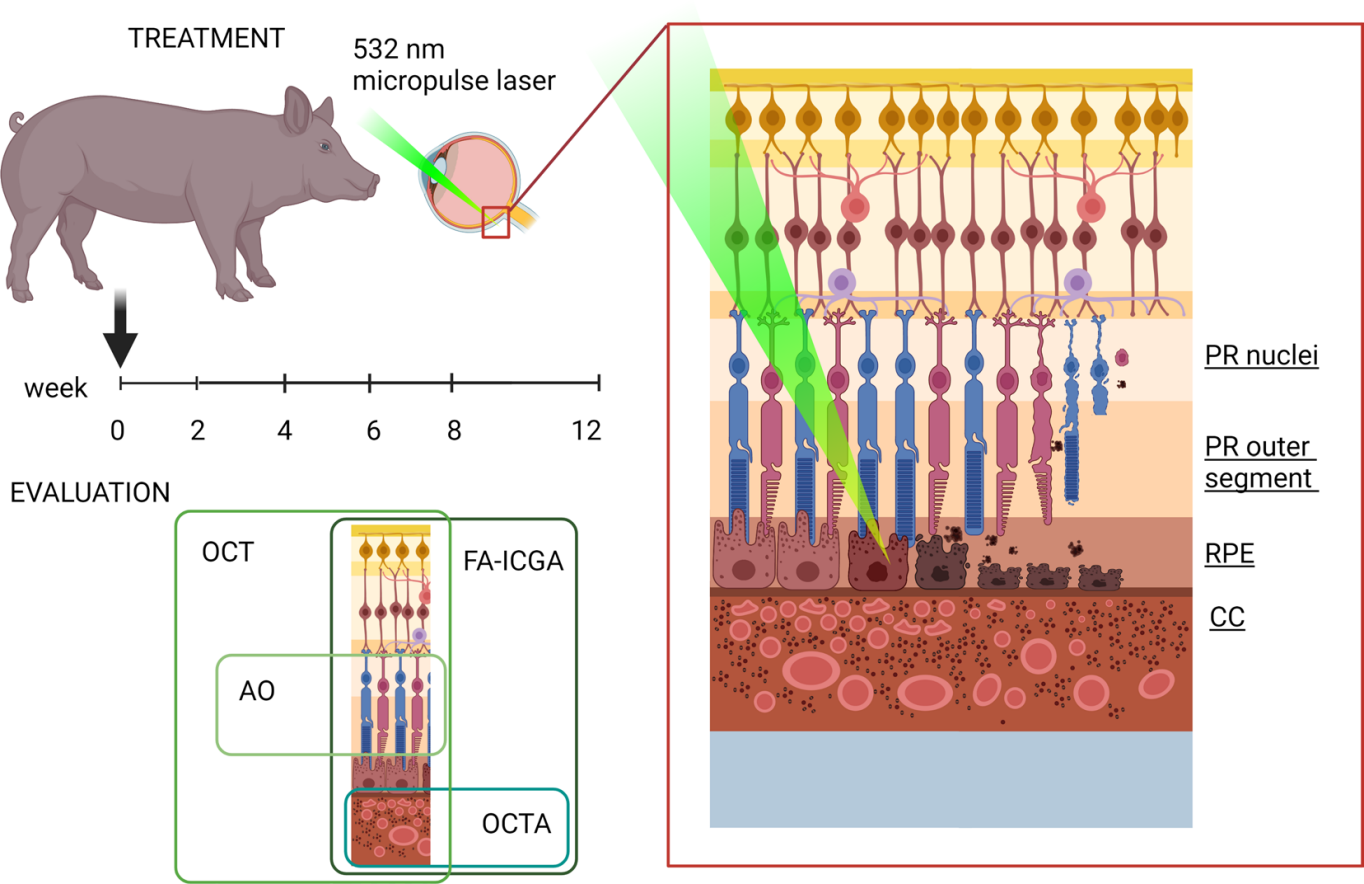
Schematic of the effect of laser injury on RPE ablation followed longitudinally using clinically relevant imaging modalities. A 532 nm micropulse laser, with a DC ranging in power from 1% to 3% was used to ablate RPE cells. Live imaging of laser-damaged eyes was performed using optical coherence tomography (OCT) to evaluate retina structure; fluorescein angiography (FA) and indocyanine green angiography (ICGA) to evaluate choriocapillaris and retinal vasculature leakage, atrophy, and proliferation; optical coherence tomography-angiography (OCTA) to evaluate choroidal vasculature confluency; and adaptive optics (AO) to image cone PRs at a single-cell resolution. Images were produced using BioRender software with publication license OT25AR5EPP.

We hypothesized that, by modulating the laser power, heat will dissipate from the RPE to adjacent PR and CC layers, allowing us to selectively damage different cell types, creating a tunable large-animal model of outer retinal degeneration (Figure 1). Seven Yucatan minipigs were enrolled in the study and treated with 4 different laser duty cycles (DC; 1%, 1.5%, 2%, and 3%) (Table 1 and Table 2). Both eyes were used for the laser injury, and each animal received multiple laser treatments per eye (3 animals received 3 treatments per eye; 4 animals received 2 treatments per eye), allowing us to reduce the total number of animals needed for the study (Supplemental Table 1; supplemental material available online with this article; https://doi.org/10.1172/jci.insight.157654DS1). Approximately 25 squares composed of 7 ***×*** 7 circular spots, with a spot size of 200 μm diameter, were aligned to cover a retinal area of approximately 1 cm^2^ (Supplemental Video 1). After the laser treatment, animals were longitudinally evaluated using clinically relevant imaging modalities, OCT, OCTA, FA-ICGA, and AO, to determine morphological and functional changes in the retina (Figure 1 and Tables 1 and 2). FA and OCT performed immediately after the laser treatment confirmed the location of laser damage and lacking interaction between different laser regions in the same eye (Supplemental Figure 1). Animals were evaluated at 2 weeks after laser for all DCs, and at 4, 6, 8, and 12 weeks for a 1% and 1.5% DC laser. Significant outer and inner retina, as well as choroid damage, was observed at 2 weeks with 2% and 3% DC laser. Since our aim was to create a model of outer retinal degeneration with preservation of the inner retina, we abandoned 2% and 3% DC laser for the long-term evaluation to further reduce animal numbers needed for this study.

### Tunable disruption of the outer blood retinal barrier.

**Figure 2:**
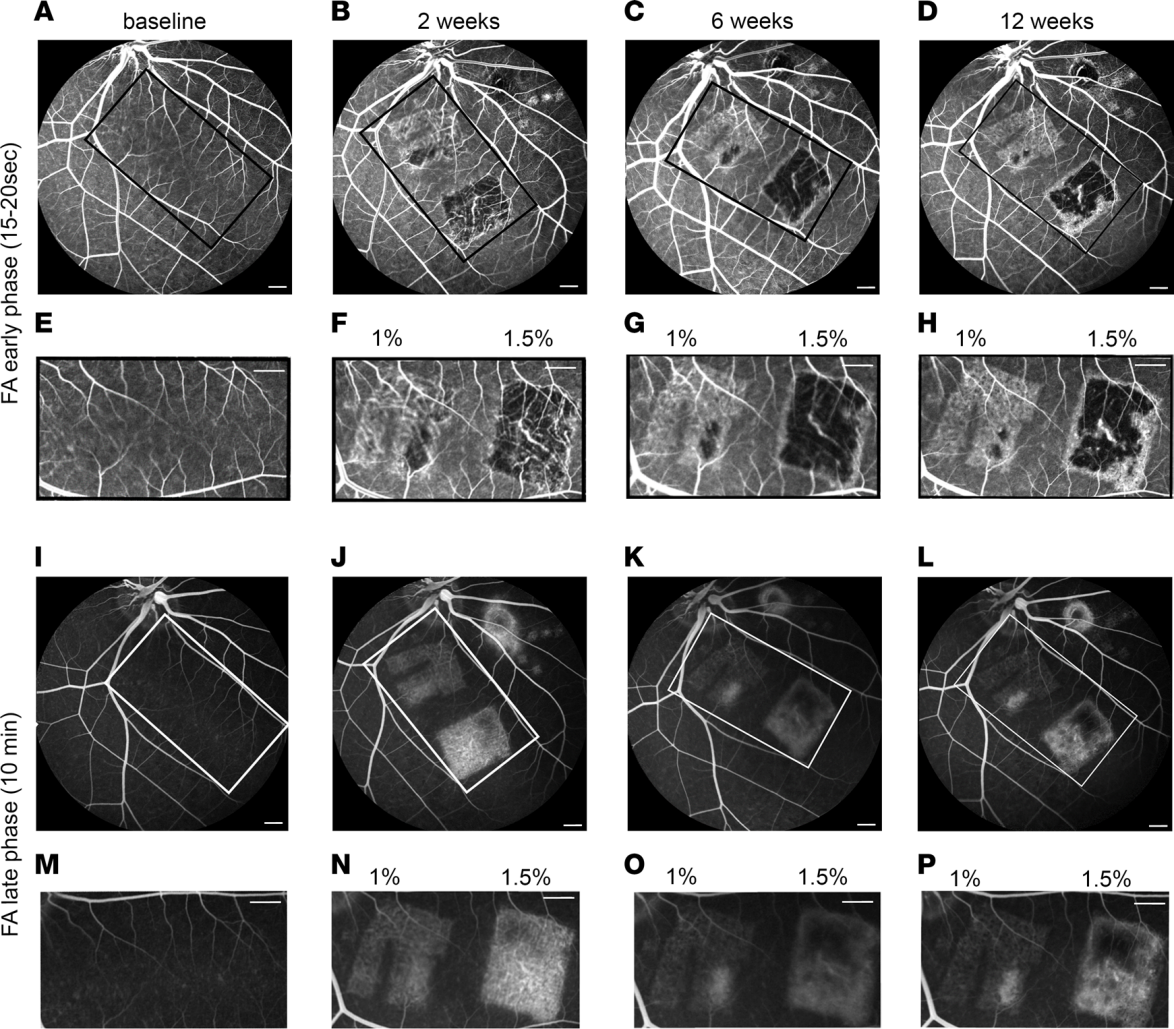
Fluorescein angiography (FA) of laser injured pig retina. (**A**–**P**) Early (**A**–**H**) and late (**I**–**P**) phase FA images of the full fundus (**A**–**D** and **I**–**L**) and higher magnification of the lasered area in the visual streak (**E**–**H** and **M**–**P**) for baseline (**A**, **E**, **I**, and **M**), 2 weeks (**B**, **F**, **J**, and **N**), 6 weeks (**C**, **G**, **K**, and **O**), and 12 weeks (**D**, **H**, **L**, and **P**) past 1% and 1.5% duty cycle (DC) laser treatment of the pig eye. Blue arrowheads mark large choroidal vessels visible through damaged RPE and choriocapillaris. Scale bars: 500 μm.

To assess the integrity of CC and the outer blood retinal barrier (oBRB), we evaluated early (15–20 seconds) and late (10 minutes) phases of FA at weeks 2, 6, and 12 (representative images shown in Figure 2). The 2-week follow-up showed 1% DC laser caused RPE atrophy, allowing CC to be visible on FA (Figure 2, A, B, E, and F). This RPE atrophy stayed stable and visible at the 6-week and 12-week follow-ups (Figure 2, C, D, G, and H). In contrast, the 1.5% DC laser led to atrophy of both RPE and CC, as characterized by hypofluorescence in the CC region and visibility of large stromal choroidal vessels, known as transmitted fluorescence or window defect (28). Injury induced by 1.5% DC laser also stayed stable until the 12-week terminal follow-up time point (Figure 2, F–H).

In the late FA phase, the fluorescein, which leaked through damaged CC, accumulated in the sclera and was visible through the damaged RPE both in 1% and 1.5% DC lasers (Figure 2, I–P). Fluorescein hyperfluorescence was higher after 1.5% DC as compared with 1% DC laser treatment at all the analyzed time points, further confirming the higher oBRB damage with 1.5% DC laser (Figure 2, I–P). After 1% DC laser injury, the Bruch’s membrane hyperfluorescence faded progressively and was barely visible at 12 weeks, suggesting a partial recovery of the oBRB. In contrast, the hyperfluorescence after 1.5% DC was observed at the 12-week terminal time point, suggesting more stable damage to the oBRB; we observed comparable damage to the oBRB complex 2 weeks after 2% and 3% DC laser (Supplemental Figure 2, A and B). However, unlike 1% and 1.5% DC laser powers, both 2% and 3% DC laser powers led to shrinkage of larger choroidal vessels, suggesting further undesired atrophy of deeper choroidal tissues.

### Quantitative assessment of CC degeneration by OCTA.

**Figure 3:**
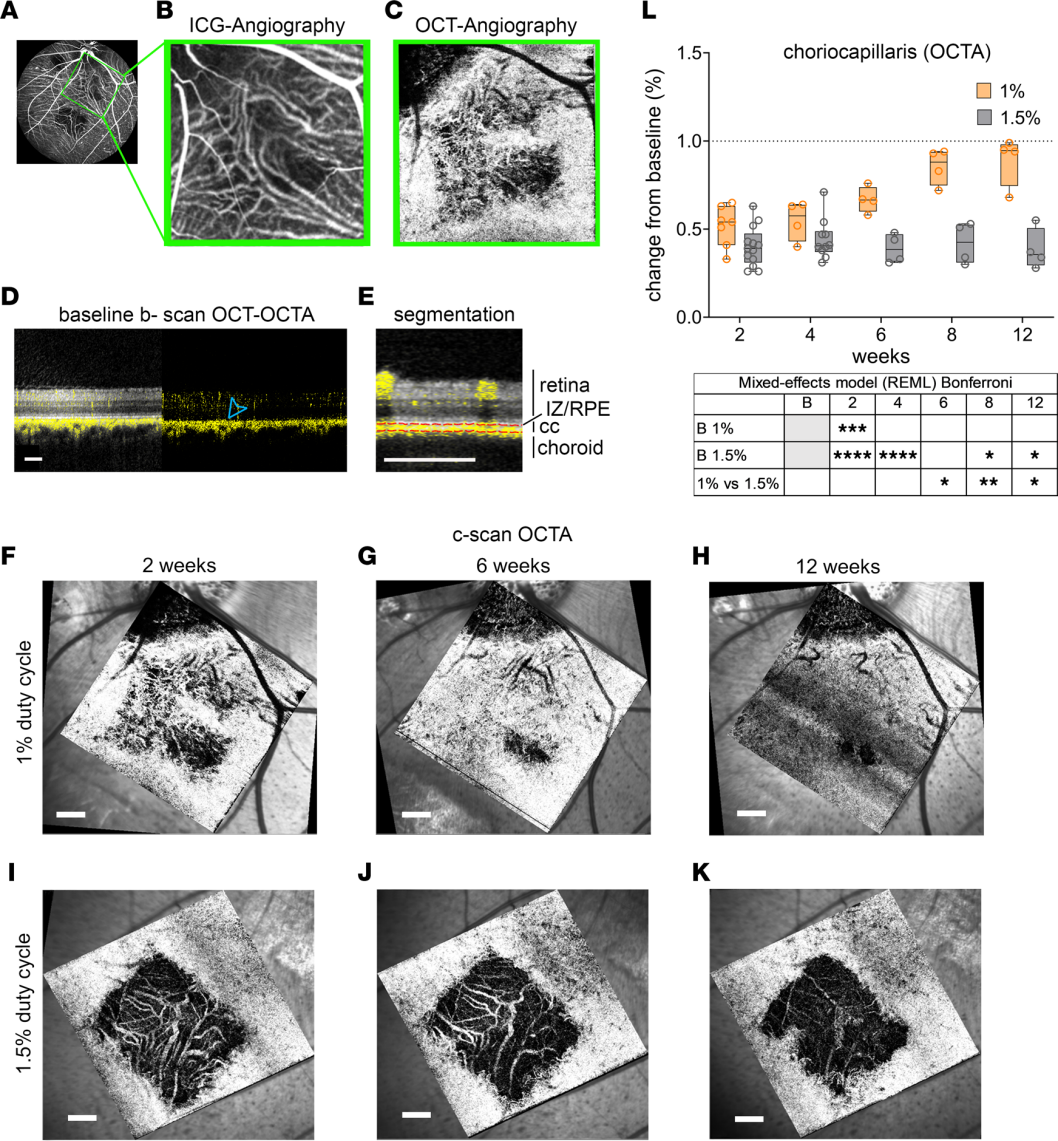
OCTA segmentation of choriocapillaris. (**A**–**C**) Simultaneous evaluation of 2 weeks 1% DC laser lesion (**A**) by ICGA (**B**) and OCTA (**C**). (**D** and **E**) Serial OCTA scans are projected as pseudo-color yellow signal; blue arrowhead shows margin of choriocapillaris as a continuous yellow signal. (**E**) OCTA signal was segmented into the retinal capillaries, choriocapillaris (CC), and choroid vasculature (red dotted lines); IZ/RPE layer was used as a landmark to define retinal from CC vasculature. (**F**–**K**) The choriocapillaris signal was represented in C-scan (en face) OCTA images of 1% duty cycle laser lesion (**F**–**H**) and 1.5% duty cycle laser lesion (**I**–**K**) shown at 2-, 6-, and 12 weeks past the laser lesion. (**L**) The graph depicts the pixel gray value changes in percent in CC density from baseline to 12 weeks as seen in en face images. Box and whiskers represent minimum to maximum, 25th and 75th percentile, median, and single values. Data were analyzed with mixed-effect model (REML) and Bonferroni multiple-comparison test. *n* = 13, 7, 4, 4, 4, 4 eyes for 1% DC at baseline, 2 weeks, 4 weeks, 6 weeks, 8 weeks, and 12 weeks, respectively; *n* = 13, 10, 10, 4, 4, 4 for 1.5% DC at baseline, 2 weeks, 4 weeks, 8 weeks, and 12 weeks, respectively. *P* values are reported as *P < 0.05; **P < 0.005; ***P < 0.0005; ****P < 0.0001. Scale bars: 500 μm

OCTA visualizes vasculature by detecting motion contrast of moving blood cells and can even evaluate subclinical alterations in CC (29, 30). Figure 3, A–C, shows a comparison between ICGA imaging (Figure 3, A and B) and CC segmented OCTA (Figure 3C) of the same retinal region, revealing higher resolution of OCTA. The differences between repeated B-scans acquired in the same location (caused by moving RBCs) revealed the presence of functional vasculature with a signal that was overlaid in yellow pseudo color. Continuous signal overlaid to the basal side of RPE represents the CC (Figure 3D, blue arrowhead). After the laser treatment, the CC signal was interrupted, suggesting CC damage (Supplemental Figure 3, blue arrow). Segmentation (Figure 3E, red dotted lines) of each B-scan was combined to create a C-scan that revealed CC damage in the lasered retina (Figure 3, F–K). After 1% DC laser, the CC signal partially recovered, starting from the borders of the lesion (Figure 3, F–H). In comparison, after 1.5% DC laser, CC did not recover (Figure 3, I–K). To quantify CC degeneration, we calculated gray pixel value in C-scans. Each lasered area value was normalized to the gray value of an untreated area within the same image (Supplemental Table 2). Quantification confirmed the initial CC degeneration after laser and recovery starting at 6 weeks only in the areas treated with 1% DC laser and not with 1.5% DC laser (Figure 3L). CC was segmented and quantified after 2% and 3% DC up to 2 weeks after lesion; however, it showed complete degeneration of the tissue (Supplemental Figure 4, A–C). Overall, OCTA quantification showed that 1.5% DC laser and not 1% DC laser was able to cause long-lasting damage to the CC.

### Qualitative and quantitative assessment of OCT to determine outer retinal damage.

**Figure 4:**
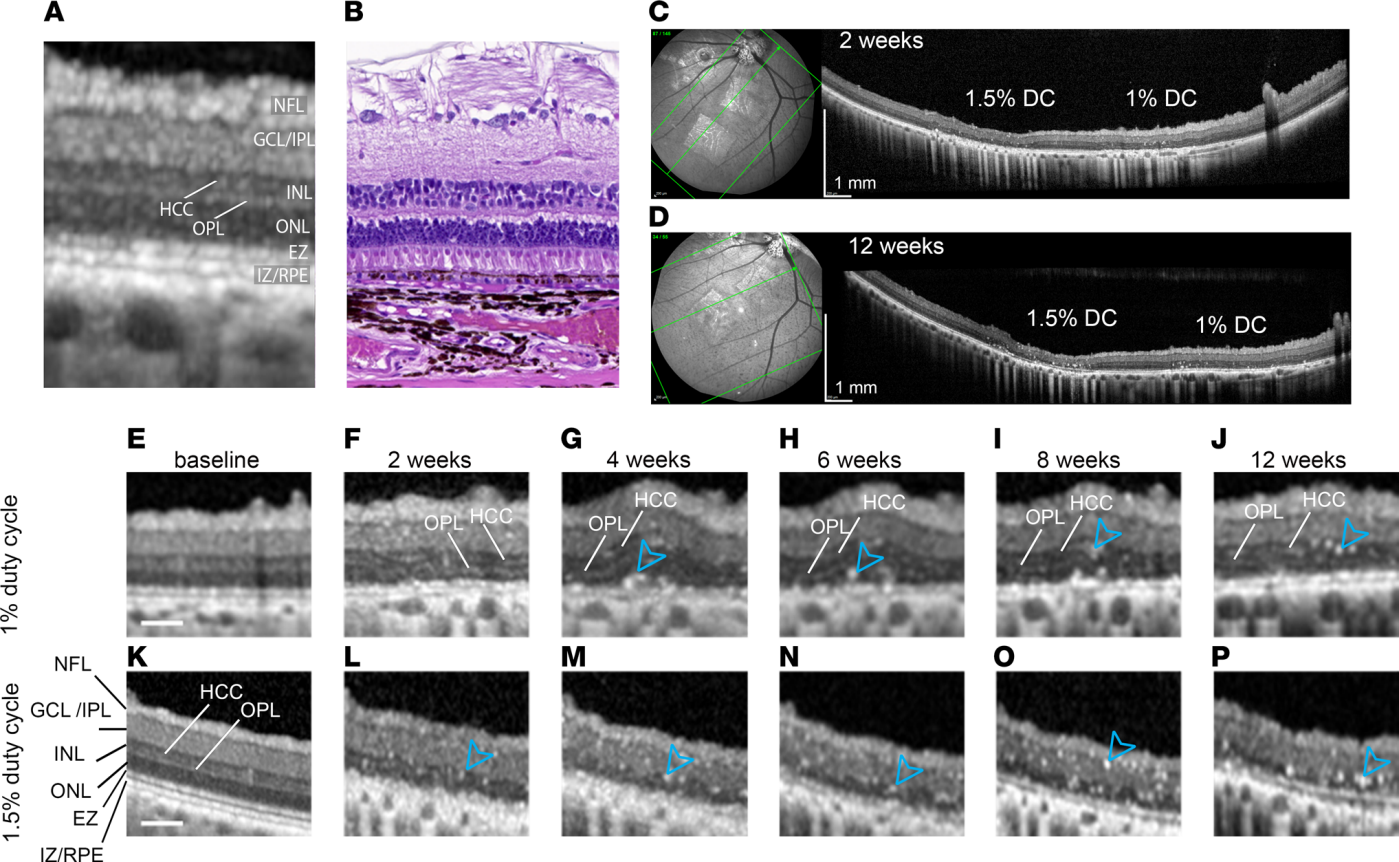
Qualitative OCT evaluation of the lasered retina. (**A** and **B**) Comparison between OCT (**A**) and histology (**B**) of the pig retina identifying the neuron fiber layer (NFL), ganglion cell layer (GCL)/inner plexiform layer (IPL), inner nuclear layer (INL), horizontal cells cytoplasm layer (HCC), outer plexiform layer (OPL), outer nuclear layer (ONL), ellipsoid zone (EZ), and interdigitation zone (IZ)/retinal pigment epithelium (RPE). (**C** and **D**) Fundus image and correspondent OCT scan at 2 weeks (**C**) and 12 weeks (**D**) after 1% and 1.5% DC laser treatment. (**E**–**Q**) Higher-magnification OCT images of laser lesions of the pig retina from 1% laser duty cycle (**F**–**K**) and 1.5 % laser duty cycle (**M**–**Q**); baseline (**E** and **L**), 2 weeks (**F** and **M**), 4 weeks (**G** and **N**), 6 weeks (**H** and **O**), 8 weeks (**J** and **P**), and 12 weeks (**K** and **Q**) after the laser injury. Scale bars: 100 μm (**A** and **B**), 1 mm (**C** and **D**), and 50 μm (**E**–**Q**). Arrowheads in G-J and L-P mark hyperreflective foci postlaser damage.

**Figure 5:**
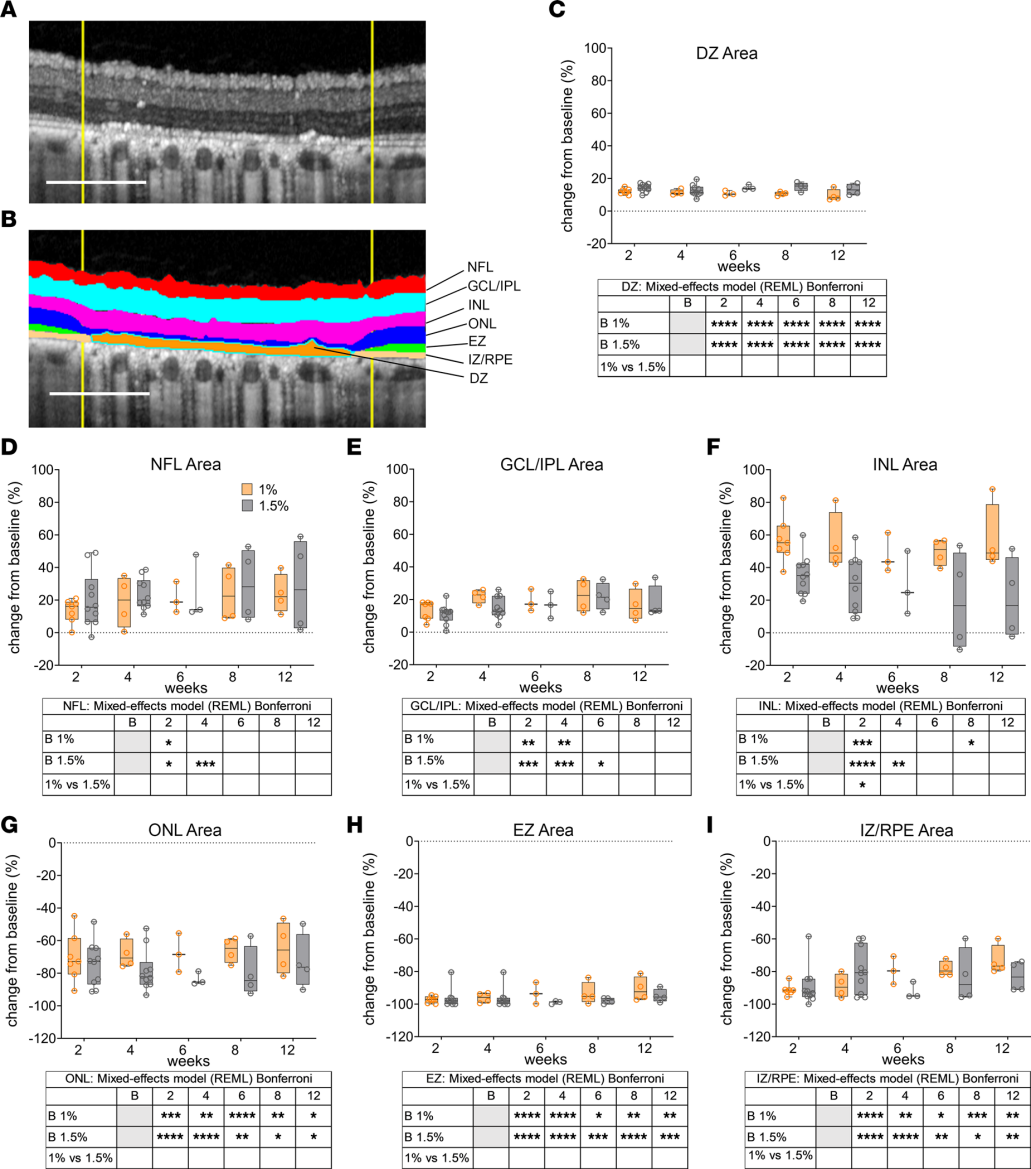
Segmentation and quantification of OCT data. (**A** and **B**) Representative image of injured retina before (**A**) and after segmentation (**B**). (**C**–**I**) Quantification of OCT images from 1% (orange line) and 1.5% (black line) duty cycle laser–injured retina based on manual segmentation of different retinal layers. Percent change of the area of the damaged zone (DZ), the neuron fiber layer (NFL), ganglion cell layer (GCL)/inner plexiform layer (IPL), inner nuclear layer (INL), outer nuclear layer (ONL), ellipsoid zone (EZ), and interdigitation zone (IZ)/retinal pigment epithelium (RPE) in 1% and 1.5% duty cycle laser–treated retinas weeks 2–12 after laser injury compared with the baseline. OCT, optical coherence tomography; HCC, horizontal cells cytoplasm layer; OPL, outer plexiform layer. (**D**–**I**) Box and whiskers represent minimum to maximum, 25th and 75th percentile, median, and single values. Data were analyzed with mixed-effect model (REML) and Bonferroni multiple -comparison test. Three OCT scan per eye were analyzed. *n* = 7, 4, 3, 4, 4 eyes for 1% DC at 2 weeks, 4 weeks, 6 weeks, 8 weeks, and 12 weeks, respectively; *n* = 10, 10, 3, 4, 4 for 1.5% DC at 2 weeks, 4 weeks, 8 weeks, and 12 weeks, respectively (**C**–**I**). *P* values are reported as *P < 0.05; **P < 0.005; ***P < 0.0005; ****P < 0.0001.

To evaluate the effect of different DC laser on pig retinal layers, we used spectral-domain OCT (SD-OCT). The correlation between SD-OCT and histology in the pig model was previously described and validated (31). Following this description, we segmented and visually correlated 6 retinal layers: nerve fiber layer (NFL), ganglion cell layer and inner plexiform layer (GLC/IPL), inner nuclear layer (INL), outer nuclear layer (ONL), ellipsoid zone (EZ), and interdigitation zone/RPE complex (IZ/RPE) to histology sections (Figure 4, A and B). After the laser treatment, all retinal structures that appeared disorganized with unidentifiable boundaries were segmented and included in a layer that we called damaged zone (DZ). Visual inspection of OCT images revealed the presence of a hyperreflective band, previously described by Xie et al., formed by the abondance of cytoplasm of the horizontal cells (called the HCC layer) (31). HCC layer was not segmented or quantified but was used as a point of reference during segmentation to assess the INL integrity. For quantification of the ONL, we used the outer plexiform layer (OPL) as the boundary between ONL and INL (Figure 4, A and B). Comparison of multiple lasered regions within the same eye further confirmed the absence of interference between different lasered areas; no variability in laser outcome was noted, whether or not a strong or a mild DC laser was used in the adjacent lasered region (Figure 4, C and D, and Supplemental Figure 5). Qualitative analysis of SD-OCT images suggested increased thickness of the inner retina, likely caused by edematous changes immediately after the laser injury — as suggested previously (32) — and by retinal remodeling at later time points (33). OPL and HCC layers were visible for all 12 weeks after 1% DC laser (Figure 4, F–K) but were not visible after 1.5% DC laser injury (Figure 4, K–P) or 2% and 3% DC (Supplemental Figure 6, A–D), suggesting that DC laser higher than 1% disrupted INL organization, even though its thickness was maintained. The EZ layer (corresponding to the mitochondria-rich PR inner segments) (31) was severely damaged by all DC laser (Figure 4, E–J and K–P, and Supplemental Figure 6, A–D), suggesting damage to the inner segments of the PRs. Expectedly, the IZ/RPE layer, corresponding to the junction between RPE and PR outer segments (31), was also severely damaged by all laser powers (Figure 4, E–J and K–P, and Supplemental Figure 6, A–D). For 1% DC, a hyperreflective layer corresponding to the IZ/RPE layer was visible but looked disorganized at all the analyzed time points, whereas after 1.5% DC, the disorganized hyperreflective layer appeared only after the 6-week time point (Figure 4, E–J and K–P). This disorganized hyperreflective layer and all abnormal areas that could not be assigned to a specific retinal layer were included in the DZ (Figure 5, A–C). After 1% and 1.5% DC laser treatment, the DZ was confined in the outer retina, while after 2% and 3%, it extended to the inner retina. Hyperreflective foci (HRF) (Figure 4, G–J and L–P, and Supplemental Figure 6, B–D, blue arrows) were visible throughout the entire retina after 1.5%, 2%, and 3% DC laser and in the INL after 1% DC, suggesting clumps of dead cells and/or immune cells that likely are removing cell debris. After segmentation, the area of each retinal layer was calculated; normalized results were graphed in Figure 5, C–I, for 1% and 1.5% DC and in Supplemental Figure 6, E and F, for 2% and 3 % DC laser. Raw pixel and normalized values are reported in Supplemental Tables 3–5. Both 1% and 1.5% DC laser injury caused swelling in NFL and GCL/IPL layers, ranging from 13.42% ± 10.60 to 29.33% ± 22.52 compared with untreated retinas (Figure 5, D and E). The INL was, however, differently affected by 2 DC lasers; at 2 weeks after treatment, 1% DC laser led to 57.04% ± 19.80 (average ± SD) swelling, while 1.5% DC caused 35.42% ± 15.71% swelling compared with the baseline. The swelling was stable for all 12 weeks for 1% DC laser, but it decreased for 1.5% DC laser, with a difference from baseline of 57.47% ± 47% and 20.68% ± 35.97% for 1% DC laser and 1.5% DC laser, respectively (Figure 5F). The outer retina (ONL, EZ, and IZ/RPE) was heavily damaged in both 1% and 1.5% DC laser. ONL thickness was reduced after both 1% and 1.5% DC laser, and at 12 weeks, the difference from baseline was –64.96% ± 26.10% for 1% DC and –73.13% ± 16.63% for 1.5% DC laser (Figure 5G). Quantification of the EZ layer showed a reduction of over 90%; the difference from baseline at 2 weeks was –97.26% ± 3.69% and –98.48% ± 2.05%, while at 12 weeks, it was –90.85% ± 10.66% and –94.92% ± 4.49% for 1% DC and 1.5% DC lasers, respectively (Figure 5H). Quantification of the recognizable IZ/RPE layer confirmed the visual observations. The difference from baseline at 2 weeks was –91.24% ± 6.23% and –89.35% ± 11.45%, while at 12 weeks, it was –73.21% ± 14.24% and –82.93% ± 11.00% for 1% DC and 1.5% DC lasered area, respectively (Figure 5I). For 2% DC and 3% DC lasers, the damage to the retinal layers was more severe as compared with 1% and 1.5% DC laser (Supplemental Figure 6, E and F). The ONL was reduced by 79.12% ± 15.77% and 86.31% ± 13.62% for 2% and 3% DC laser, respectively (Supplemental Table 5). EZ and IZ/RPE layers were completely absent. The inner retina was severely swollen and showed abundant HRF, suggesting severe cell clumps/immune cell invasion and degeneration of the entire retina by these higher laser powers (Supplemental Figure 6, B and D). Overall, OCT also confirmed selective and tunable damage to different retinal layers by laser power of different DC.

### AO-based quantification confirms cone PR degeneration.

**Figure 6:**
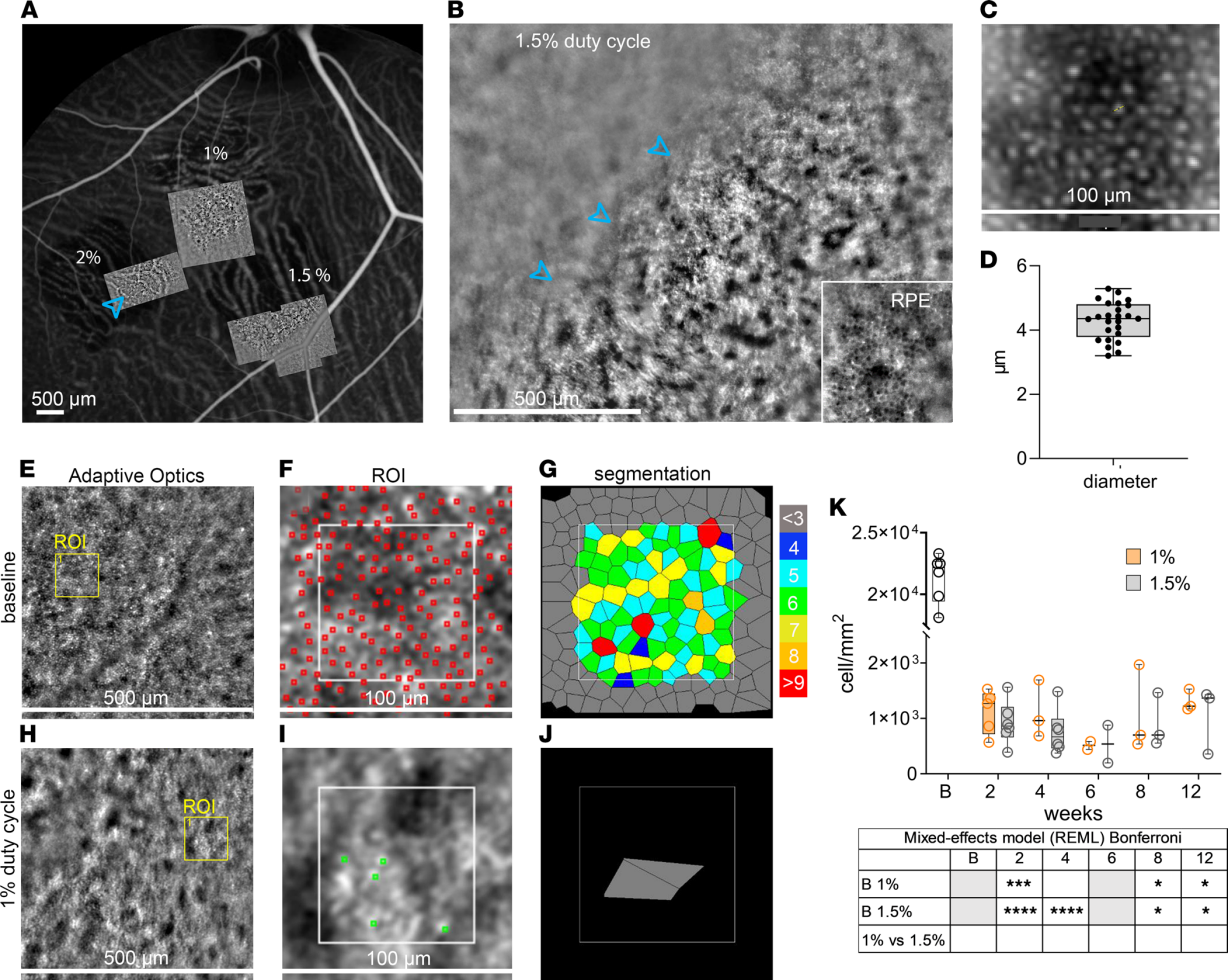
Adaptive optics–based (AO-based) quantification of cone photoreceptor cell damage in laser-injured pig retinas. (**A**) En face view of pig fundus with aligned AO montage images corresponding to 1%, 1.5%, and 2% DC laser damage. The cyan arrowhead points to the choroidal vessels visible through the damaged retina. (**B**) Higher magnification of an AO imaged area in 1.5% duty cycle laser–injured retina; cyan arrowheads are pointing at the margin of the lasered retina. Detail of pig RPE imaged outside the visual streak. (**C** and **D**) Cone diameter calculated on untreated pig retina. (**E**–**J**) Representative AO image of baseline (**E**) and 1% duty cycle laser injured retinal area (**H**); identification and segmentation of photoreceptors in baseline (**F** and **G**) and 1% duty cycle laser–injured retinal area (**I** and **J**); and red dots marking cone PRs used for automated quantification at baseline, with green dots indicating adjusted quantification in lasered areas. (**K**) Photoreceptor cell density per mm^2^ compared with baseline images in 1% (orange) and 1.5% (gray) laser–injured pig retinas at 4, 8, and 12 weeks after injury. Scale bars: 500 μm (**A**, **B**, **E**, and **H**) and 100 μm (**C**, **F**, and **I**). (**D** and **K**) Box and whiskers represent minimum to maximum, 25th and 75th percentile, median, and single values. Data were analyzed with mixed-effect model (REML) and Bonferroni multiple-comparison test. Five regions of interest (ROI) in 3 images for each eye were analyzed. *n* =25 eyes (**D**); *n* = 6, 5, 3, 3 for 1% DC at baseline, 2 weeks, 4 weeks, 8 weeks, and 12 weeks, respectively; 6, 6, 3, 3 for 1.5% DC at baseline, 2 weeks, 4 weeks, 8 weeks, and 12 weeks, respectively (**K**). The 6-week time point was excluded from quantification; 1% and 1.5% DC were shown as *n* = 2 eyes. *P < 0.05; ***P < 0.0005; ****P < 0.0001.

To better understand the progression of cone PR degeneration and correlate our data to outer retina diseases (34–36), we performed longitudinal analysis on laser-injured pig retina with an AO flood–illuminated retinal camera. Using FA fundus images, we mapped retinal vessels and marked laser-injured regions (Figure 6A). RPE disruption made the choroidal vessels visible in AO as hyperreflective areas, supporting identification of the damaged retina (Figure 6A, cyan arrowhead). Lasered area margins were well defined, and the lasered retina was visible in AO on a different focal plan compared with the adjacent uninjured retina (Figure 6B, cyan arrowheads pointing the margin of the lasered retina). Outside of the visual streak, the RPE monolayer was also visible (Figure 6B). Pig cone PRs in the uninjured visual streak appeared as round hyperreflective dots (HRDs) with an average diameter of 4.29 ± 0.58 μm and uniform distribution (Figure 6, C and D). After the laser injury, the shape and distribution of HRDs became irregular (Figure 6, B and H), similar to previous findings of cellular debris associated with ONL loss in patients with retinitis pigmentosa (37), suggesting cone PR degeneration in our model.

To quantify differences in cone PR morphology and degeneration before and after the laser treatment, we segmented 5 regions of interest (ROI) for each image (Figure 6, E–G) and analyzed cone PR density, number of neighbors, and inter cone PR spacing, as suggested previously (38, 39). At baseline, the number of cone PR neighbors in the pig visual streak averaged 5.4 ± 1.7; the frequency of cone PRs with 5 neighbors was 20.7%, with 6 neighbors was 22%, and with 7 neighbors was 15.4%. Spacing between cone PRs, calculated from center to center of adjacent cells, averaged 6.3 ± 3.7 μm. However, after the laser treatment (both 1% and 1.5% DC laser), the number of neighbors and cone PR spacing was not possible to calculate for laser-treated areas (Figure 6, H–J). Two weeks after laser injury, cone PR density was significantly decreased from 1,984.6/mm^2^ ± 1,522.9/mm^2^ (baseline) to 1,119.8/mm^2^ ± 809.8/mm^2^ after 1% DC laser and 1,128.7/mm^2^ ± 1,035.7/mm^2^ after 1.5% DC laser (Figure 6K). At 2 weeks after higher, 2% and 3%, DC laser, the density was drastically reduced to 895.18 ± 777.92 and 667.31 ± 587.38/mm^2^, respectively (Supplemental Figure 7). Reduction in cone PR density stayed stable for 1% DC laser and 1.5% DC laser for the 12 weeks after treatment analysis.

### Differential effect of laser power on the ONL thickness and the immune cell activation.

**Figure 7:**
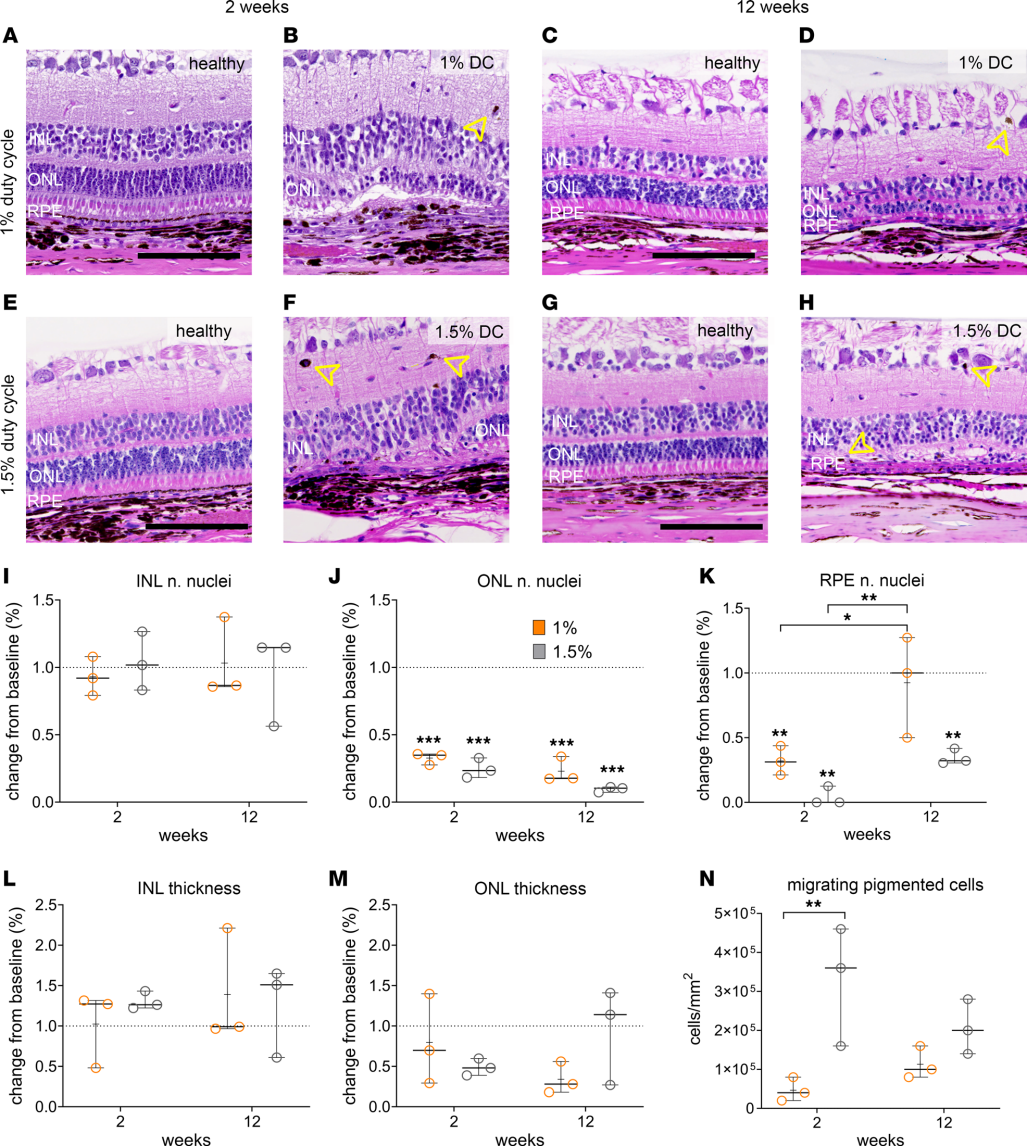
Histological evaluation of the laser injured retina. (**A**–**H**) representative images of healthy (**A**, **C**, **E**, and **G**) and laser-injured (**B**, **D**, **F**, and **H**) retina after 2 and 12 weeks of 1% and 1.5% DC laser treatments. (**I**–**K**) Nuclei number quantification and comparison between healthy and laser-treated area of the retina for INL (**I**), ONL (**J**), and RPE (**K**) at 2 and 12 weeks after 1% (orange) and 1.5% DC (gray) laser treatments. (**L** and **M**) Quantification of retina thickness for INL (**L**) and ONL (**M**) at 2 and 12 weeks after 1% and 1.5% DC laser treatments. (**N**) Quantification of migrating pigmented cells per mm^2^ of retina identified at 2 and 12 weeks after 1% and 1.5% DC laser treatments. (**I**–**N**) Box and whiskers represent minimum to maximum, 25th and 75th percentile, median, and single values. Data were analyzed by 2-way ANOVA and Bonferroni multiple-comparison test. Five slides for each eye were analyzed. *n* = 3 eyes per condition (**I**–**N**). Arrow heads in B, D, F, and H mark migratory pigmented cells *P < 0.05; **P < 0.005; ***P < 0.0005. Scale bar: 100 μm.

**Figure 8:**
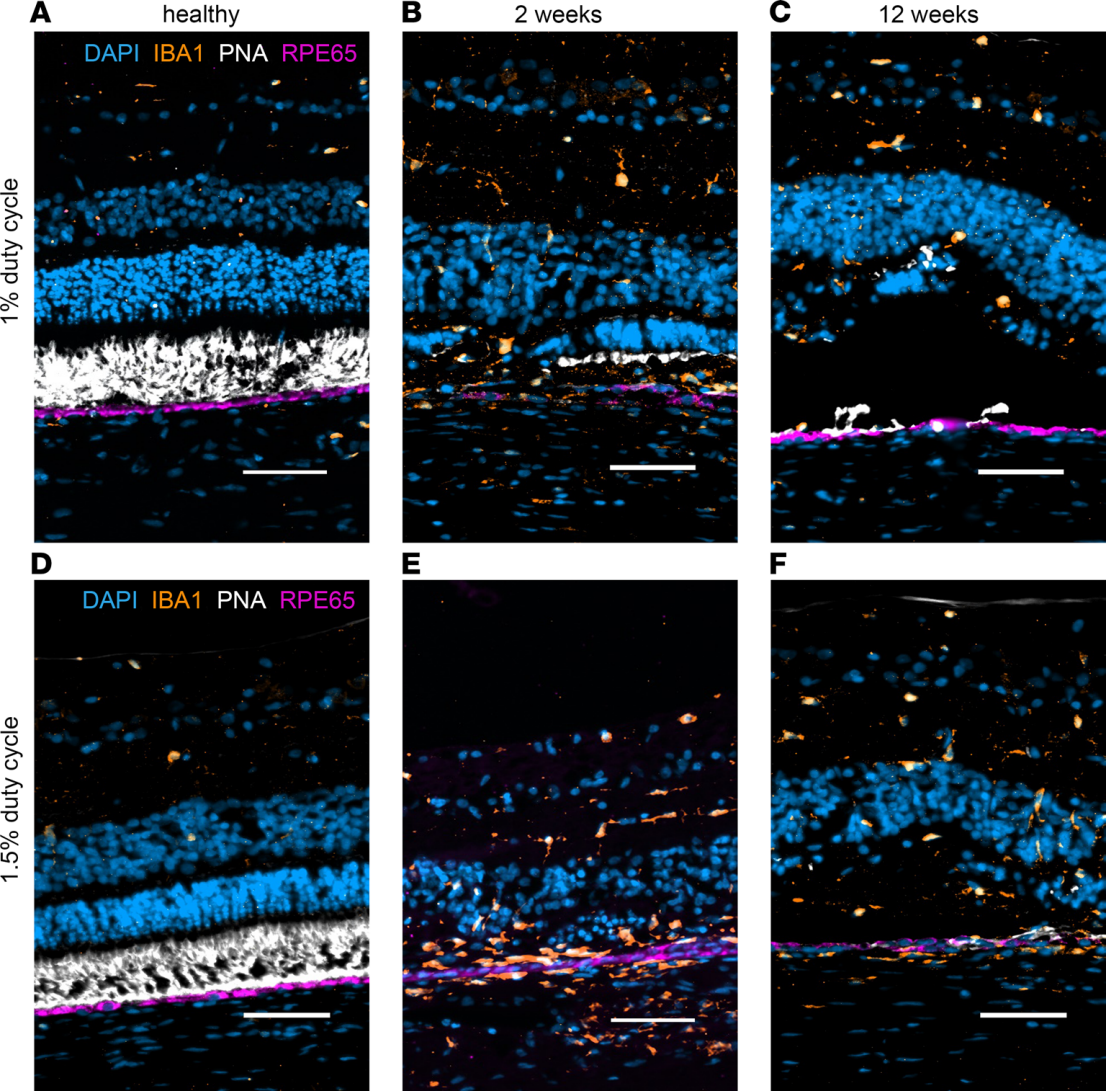
IHC confirms damage to photoreceptors and RPE and shows immune cell activation in the lasered injured retina. (**A**–**F**) Healthy retina (**A** and **D**) and 1% and 1.5% DC laser–treated retina at 2 weeks (**B** and **E**) and 12 weeks (**C** and **F**) after treatment. RPE65 labels the retinal pigment epithelium (magenta). PNA labels the cone photoreceptor outer segment (white). IBA1 labels immune cells (orange). DAPI labels cell nuclei (cyan). Scale bar: 50 μm. Two of the total eyes (1% DC *n* = 3 at 2 weeks, *n* = 5 at 12 weeks; 1.5% DC *n* = 7 at 2 weeks, *n* = 5 at 12 weeks) were fixed and processed for IHC.

At 2 and 12 weeks after laser injury, animals were euthanized and eyes were processed for histological and IHC evaluation (Figures 7 and 8). H&E staining revealed significant PR outer segment degeneration, along with some ONL disorganization in 1% DC laser (Figure 7, A–D). In contrast, both the PR outer segments and the ONL were found to degenerate after 1.5% DC, and the INL appeared disorganized with increased spacing between adjacent bipolar cell nuclei (Figure 7, E–H). Cell nuclei quantification of INL and ONL in 1% and 1.5% DC laser–treated retinas at 2 and 12 weeks after laser compared with the baseline data shows that the of number INL nuclei was not affected by the laser treatment (Figure 7I), whereas the number of ONL nuclei was significantly reduced at both time points (Figure 7J). At 1%, the DC laser reduced the number of RPE nuclei to about 30% when analyzed at the 2-week time point but cell number recovers to baseline levels by 12 weeks, suggesting proliferation of RPE from the edges of the laser lesion (Figure 7K). In comparison, 1.5% DC laser led to a more severe reduction in RPE nuclei to less than 10% when analyzed at the 2-week time point, with recovery up to 30% by 12 weeks (Figure 7K). Consistent with lacking laser effect on INL nuclei number, INL thickness was preserved until 12 weeks after 1% and 1.5% DC laser treatment (Figure 7L). In comparison, the ONL thickness was reduced by 20% after 2 weeks of 1% DC laser treatment and continued to decline until week 12, when merely 35% of the baseline thickness was detectable (Figure 7M). Expectedly, after 1.5% DC laser, the ONL thickness dropped to approximately 40% of the baseline levels; however, this drop seemed to recover closer to the baseline levels by week 12 (Figure 7M). This discrepancy in ONL nuclei number, as measured by histology, and ONL thickness, as measured by OCT, is likely due to the inability of OCT segmentation algorithm to distinguish between the ONL and fibrotic changes happening in that region of the retina by week 12 (Figure 4, K and Q, and Figure 7, D and H). Histological analysis revealed the presence of migrating pigmented cells (Figure 7, B, D, F, and H, yellow arrowheads); the number of these cells was directly proportional to the laser power (Figure 7N). Overall, histological analysis and quantification confirmed that 1% DC laser causes a temporary damage to the RPE and damage to PRs, whereas damage ensued by 1.5% DC laser is longer lasting.

Immunostaining for mature RPE marker RPE65 showed results consistent with histological analysis. There was minimal and sporadic RPE65 signal at 2 weeks after 1% DC laser that recovered slightly by 12 weeks (Figure 8, A–C). In contrast, the RPE65 signal after 1.5% DC was barely visible at week 2 or 12 after the laser injury, suggesting complete RPE ablation (Figure 8, D–F). PNA staining, marking cone PR outer segments, showed mild staining at 2 weeks after 1% DC laser but no staining at 12 weeks after 1% DC laser or 2 and 12 weeks after 1.5% DC laser, corroborating our histological analysis (Figure 8, A–F). IBA1 immunostaining confirmed the presence of immune cells in the retina after both 1% and 1.5% DC laser, with migratory cells seen in the entire retina and in the choroid after 1.5% DC laser, as compared with 1.0% DC laser, where they were confined mainly in the inner retina, suggesting higher inflammation after 1.5% DC laser, consistent with our histological analysis (40). In comparison with 1% and 1.5% DC laser, retinas treated with 2% and 3% DC laser processed 2 weeks after the laser injury showed complete degeneration of RPE, EZ, ONL layers; significant remodeling and degeneration of the INL; and presence of migratory cells (Supplemental Figure 8, A and B, yellow arrows). Nuclei quantification confirm the H&E observation of severe reduction in the nuclear count of RPE down to 20% for 2% DC and 8% for 3% DC laser, ONL down to 15% for 2% DC and 5% for 3% DC laser, and INL down to 65% for both 2% and 3% DC lasers (Supplemental Figure 8C).

## Discussion

We report the development and characterization of a large-animal model that allows tunable outer retinal degeneration. Previously, in a pig model, we demonstrated that a 532 nm laser with 1% DC laser produced a localized RPE damage and consequent PR degeneration (27). Here, we extended those findings by increasing the DC laser. At 1% DC laser power, a focal damage was seen in the RPE and PRs; at 1.5% DC laser power, damage extended to PRs, RPE, and the CC; and 2% and 3% DC laser completely atrophied all retinal layers, making these laser powers useless for developing an outer retinal degeneration model. The use of 1% and 1.5% DC lasers allows the flexibility to model numerous retinal degenerative conditions, including those affecting the RPE and PRs (intermediate to late-stage AMD and forms of RP) and those involving RPE, PRs, and the choroid (late-stage AMD/GA and choroideremia). Our model also recapitulates battlefield laser injuries that have become a growing concern with modern day warfare (7). Furthermore, this model accommodates degeneration as large as 50 mm^2^ within the visual streak of the pig retina, allowing testing of a transplant of size that would cover the entire human macula. To reduce the number of animals used in this study, we often targeted 2 or 3 different laser powers in 1 eye. Specific DC laser locations were randomly chosen and were separated by > 1 mm to avoid interference between them. Our data don’t suggest any bias or interference between different laser procedures, making this a robust approach to evaluate different types of injuries within 1 eye. In-depth characterization of the retinal and choroidal injury using clinically relevant imaging modalities including, for the first time to our knowledge, AO and OCTA combined with quantification of OCT, OCTA, and AO data further enhances the clinical relevance of this model to develop treatments for human retinal degenerative diseases. Combined, the versatility and translatability of our model allows testing a range of therapeutics targeting multiple retinal degenerative diseases.

Compared with 1% DC laser, 1.5% DC laser caused long-lasting and gradated choroid degeneration. For instance, the late-phase FA suggested a loss of the RPE barrier function in both 1% and 1.5% DC laser. In comparison, the early-phase FA showed missing CC signal only in 1.5% DC laser, exposing bright choroidal vessels from underneath (41). Interestingly, a similar phenomenon is described in geographic atrophy, with missing CC signal in early-phase FA and late-phase fluorescein accumulation visible in the choroidal stroma, underscoring the relevance of our model in studying stages of AMD (28). OCTA complemented findings by FA because OCTA uses retinal layer volumetric data segmented to separately visualize retinal capillary plexi and the CC (42, 43). OCTA C-scan quantification confirmed that CC had lasting damage in 1.5% DC laser as compared with 1% DC laser. Both FA and OCTA revealed partial recovery of the RPE barrier function from the borders of the lesion, visible as FA hyperfluorescence along the lesion margins, more prominent for 1% than 1.5% DC laser. A potential reason for this recovery in our data is suggested by combining FA with OCTA and histology that 1% DC likely disrupted RPE tight junctions in some areas without significant cell death, while 1.5% laser caused significant RPE cell death and CC atrophy (44). Damaged RPE tight junctions likely recovered faster on the edges providing regenerating signals to the CC. That recovery, however, wasn’t sufficient in the middle of the lesion to rescue CC or the PRs. Another potential reason for this recovery is that, after RPE ablation, the relatively healthy RPE cells at the margins of the lesion migrate in an attempt to repopulate the damaged area, which can also be seen at the margins of lesion in 1.5% DC laser (45). Finally, this recovery may be due to proangiogenic signals from relatively healthy and uninjured RPE cells on the border outside the laser lesion. Overall, our data show how modulating the DC of a 532 nm laser causes an irreversible or reversible RPE and CC damage. We anticipate that, in using a different laser wavelength, one can induce specific injuries in different parts of the retina, depending upon the wavelength susceptibility of that region, further highlighting the versatile and nongenetic retinal degeneration approach used in our study. Interestingly, the relationship between RPE atrophy and the consequent CC degeneration is also described in patients with GA (28, 46), making this model highly relevant to further studying RPE-CC interactions.

Similar to the CC, PR degeneration may be a consequence of RPE atrophy (47). OCT revealed severe thinning of the outer retina layers, RPE-PR interface (ONL, EZ, and IZ/RPE), and swelling of the inner retina layers, reflecting edema (32). One limitation of OCT, as compared with histology, is that it likely overestimated the outer retinal degeneration. This is because OCT quantification was only feasible in portions of the retina that could be segmented (for instance, DZ could not be segmented), whereas, in histology, all layers were identifiable at the single-cell resolution. This comparative analysis of OCT and histology highlights the need for complementary outcome variables when studying retinal degenerations and the effect of therapeutic modalities.

Our OCT data suggest higher INL, NF, and IPL thickness after both 1% and 1.5% DC laser. We hypothesize that this increased thickness is likely associated with a postlaser inflammatory response; our hypothesis is supported by the presence of HRF seen by OCT in both laser powers. We further hypothesize that HRFs are caused by laser-induced aggregates of dead cells, melanin aggregates secondary to RPE disruption, or aggregates of inflammatory cells that are trying to clear cell debris (40, 48, 49). The observation of HRF by OCT was consistent with migrating pigmented cells observed in H&E-stained sections. Immunostaining also revealed punctate IBA1 signal 2 and 12 weeks after 1% and 1.5% DC laser injury, suggesting immune/retinal microglial cells as the possible cause for HRF, as suggested previously in rats treated with a 100 ms pulse and 532 nm laser (50) and in the pig retina after iodoacetic acid–induced PR degeneration (13).

Cone PR morphology and distribution are 2 main identifying characteristics in AO images (51–53). Pig cone PR diameter in our study was within the range of previously reported human cone PR diameter (3.0 ± 0.4 μm to 8.2 ± 0.6 μm; mean ± SD) (54, 55). In our data, the cone PR density at the baseline was consistent with Huckenpahler (51) and comparable with spacing and density of human cones PRs imaged at 4° eccentricity using the same AO system used in our study (52). It is worth noting that both the RTX1 AO system used in our study and techniques like adaptive optics scanning laser ophthalmoscope (AOSLO) underestimate the cone PR density by 20%–30% as compared with histological analysis (51, 56). Furthermore, laser injury prevented proper segmentation of cone PRs due to low density, precluding calculations of cone PR spacing and number. Similar concerns have been raised for AO analysis of retinitis pigmentosa and choroideremia patients’ eyes (37, 57), suggesting a limitation of AO assessment of low-density cone PR areas.

Our data support the tunable nature of this laser-induced model for retinal/RPE/choroidal injury, allowing us to mimic early to advanced disease stages and providing the possibility of testing cell and gene therapies, as well as retinal prostheses, of a clinical dose that can cover the entire human macula. The laser injury model does has some limitations. (a) The thermal damage induced by the laser causes an inflammatory reaction that may aggravate retinal degeneration and interfere with the tested therapy. The inflammatory response can be reduced by administering antiinflammatory and immunosuppressive drugs (27). (b) The extent of laser injury is dependent on RPE pigmentation, resulting in variable results in animals with low or uneven pigmentation. We resolved this limitation by using Yucatan minipigs, a highly pigmented swine breed and by defining the laser power using the minimal visible threshold. Relatively low variability of our results is supported by high consistency in our OCT and OCTA data. (c) The lowest DC laser used in our work was 1%. This laser power still caused damage to both RPE and PRs, even though the RPE damage is not long lasting. It is anticipated that DC laser lower than 1% will result in RPE-only damage, sparing PRs. However, this is currently technically not feasible. Our approach used minimal visible threshold that uses a color change of the retina, allowing us to visualize the laser spot and manually move the laser to the adjacent location. Equipment allowing automated movement of the laser probe may be required for laser power below 1% DC laser.

Overall, our model has several advantages compared with inducible models of retinal degeneration that use systemic, intravitreal, or subretinal injection of different chemicals (13, 58). The damage in the laser model can be controlled and modulated for each subject to create a predictable grade of degeneration; multiple areas of the retina can be damaged with different laser intensities and different laser wavelengths that may target different parts of the eye. Laser-induced cell degeneration is rapid and happens over weeks as compared with a mutation-induced cell death that may take years. Laser damage can be obtained at any age of animal, reducing the cost and the length of the study compared with transgenic models that may need to be kept for months or years (21, 22). Compared with transgenic models, the laser model can be used to study therapeutical approaches for multiple types of retinal degeneration. In particular, the laser pig model is an optimal large-animal model to investigate therapeutic cell and reparative therapies and for developing surgical approaches for AMD, Stargardt macular dystrophy, and choroideremia.

## Methods

### Study design

All animals received a baseline examination, before the laser injury, including OCT, OCTA, FA-ICGA, and AO. The same set of examinations were repeated at 2, 4, 6, 8, and 12 weeks after the laser treatment. To minimize the number of animals used in this study, each eye received 2 or 3 laser treatments in different areas along the visual streak. Laser injuries were separated along the retina to ensure no interference between different laser treatments. At the end of the study, all animals were euthanized, and the eyes were collected for histological and histochemical evaluation.

### Animals

Yucatan minipigs from Premier BioSource/S&S Farms and Sinclair Research with homogeneous fundus pigmentation were enrolled in the study. Animals (castrated males, 35–45 Kg) were housed in climate-controlled rooms with wood shavings on the floor, food was administered twice a day, and water was offered ab libitum.

For imaging and laser injury, pigs were preanesthetized using glycopyrrolate (0.01 mg/kg, i.m., American Reagent Inc.); after 20–30 minutes, anesthesia was induced by i.m. administration of telazol (5 mg/kg, Zoetis) mixed with butorphanol (0.2 mg/kg, Torbugesic, Zoetis) and dexmedetomidine (0.035 mg/kg, Dexmedesed, Dechra). Animals were then moved to the operating room and intubated, and an i.v. catheter was placed. Anesthesia was maintained with isoflurane or sevoflurane using a volume/pressure combined ventilator. Pigs were positioned in dorsal decubitus in custom cradles. Water and air-warming blankets were used to maintain the body temperature. Blood pressure, heart rate, blood oxygenation, CO_2_, and temperature were monitored continuously. Sodium chloride (0.9% sodium chloride injection United States Pharmacopoeia grade [USP], Hospira) or lactated ringer (Lactated Ringers injection USP, ICU Medical) solutions were administered throughout the procedure. Pupils were dilated with tropicamide 1% (Tropicamide Ophthalmic solution 1% USP, Akorn) and phenylephrine 2.5% (phenylephrine hydrochloride ophthalmic drops 2.5%USP, Paragon Biotech). During image collection and laser injury, rocuronium (2–3 mg/kg, i.v.; rocuronium bromide injection 10 mg/mL USP, XGen) was administered as needed for relaxation of the extraocular muscles. Upon completion of imaging, an ophthalmic ointment (Neomycin and Polymyxin B Sulfates Ophthalmic ointment USP, Bausch and Lomb) was placed on the corneal surface. Ketoprofen (3 mg/kg, i.m., Ketofen 100 mg/mL, Zoetis) to reduce muscle pain related to paralytic administration. Fluorescein and ICG are administered i.v. for OCTA. Preparing for enucleation, pigs were anesthetized using the protocol outlined above, intubated, and maintained on a pressure-controlled ventilator. Following enucleation, pigs were euthanized immediately by administering B-euthanasia i.v. 1 mL per 10 lbs of body weight (euthanasia solution, VetOne). Animal’s heart rate, blood pressure, and respiration were monitored to ensure euthanasia.

### Laser injury

Laser injury technique used in this study was previously reported by Sharma et al. (27). An IQ 532 nm micropulse green laser (Iridex) equipped with TxCell technology was used. A 532 nm wavelength is efficiently absorbed by the RPE melanin. As opposed to continuous wave lasers, in micropulse mode, the exposure time is divided into pulses called DC with a short ON-time and a long OFF-time that allows tissue to cool down between pulses, thus limiting the area of tissue damage to the RPE and vicinity. In addition, TxCell technology allows for the making of confluent lesions in the treated area. Laser power was adjusted to reach the minimum visible threshold to compensate for the natural changes in the pigmentation of the pig eyes (59). Spot size of 200 μm and 330 ms of exposure time, DC of 1% (0.1 ms on to 9.9 ms off), 1.5% (0.1 ms on to 6.6 ms off), and 2% (0.1 ms on to 4.9 ms off) were used with laser power of 1800 mw (1% DC), 1600 mw (1.5% DC), and 1100 mw (2% DC). Confluent threshold (visible) lesions were performed in the area of interest. The threshold was tested as immediate whitening of the retina corresponding to the pulse location (Supplemental Video 1).

### OCT

OCT images were obtained using the Spectralis SD-OCT (Heidelberg Engineering) instrument. The device uses a broadband super luminescent diode emitting a scan beam at a wavelength of 870 nm. Confocal scanning-laser ophthalmoscopy (cSLO) scans of the fundus were acquired simultaneously with a near-infrared (near-IR) wavelength. Each fundus scan was recorded with a scan angle of 55°. Eye-tracking technology allowed for consistent volume scan locations within the retina over all time points.

During each imaging session, 3 OCT volumes were recorded for each eye: 1 radial scan (centered in the visual streak) and 2 raster scans. Raster scans were recorded parallel and perpendicular to the visual streak with a scan angle of 55° and pattern size of 55° ***×*** 45° (16.8 mm ***×*** 13.8 mm). All B-scans were obtained at 400,000 A-scans per second with a depth and transverse scale of 10.94 μm/pixel and 3.87 μm/pixel, respectively. To improve signal/noise ratio, speckle noise, and contrast, each scan was averaged over 19 ± 2 images with the automatic real-time tracking (ART) function. Radial and raster scan volumes consisted of up to 48 images and 217 images, respectively.

To quantify the effect of the laser treatment and the progression of the damaged tissue, the cross-sectional area of retinal layers was recorded in equally sized OCT scans and compared over time. Cross-sectional area measurements were preferred to A-scan (axial) thickness due to greater variation in curvatures and rotation found in laser-ablated tissue. To objectively determine 3 images (across all time points) for analysis, the boundaries of the lasered area were first determined in relation to the OCT scan volume. B-scans located at the quarter, halfway, and 3-quarter points between these 2 boundaries were exported for analysis. This was repeated for every lasered region within a given eye.

Each image was cropped 50 pixels outward of the lasered region. Outward cropping allowed for more efficient segmentation, as observers were able to visualize layers over healthy and damaged regions. Subsequently, the NFL, GCL/IPL, INL, ONL, EZ, and interdigital zone/RPE (IZ/RPE) layers were segmented using ImageJ (NIH). Segmentation masks delineating layers by color were exported in TIFF format. The excess 50 pixels on both sides of the laser boundaries were removed, and the area of each layer was quantified using custom MATLAB scripts.

### OCTA

#### OCTA imaging.

OCTA images were obtained using the Spectralis SD-OCT (Heidelberg Engineering) instrument. Each OCTA B-scan contains between 384 and 768 A-scans, and each OCTA volume contains between 256 and 512 B-scans. OCTA volumes were centered on specific laser ROIs.

#### OCTA analysis.

En face OCTA scans were exported from the HEYEX Heidelberg software in TIFF format. Retinal vasculature in each lasered region was directly compared with vasculature in an adjacent, healthy portion in the scan to account for brightness variations among scans. An experienced observer obtained the gray value from each en face scan, or the average binary pixel intensity, for as big of an area as possible in both lasered and healthy regions. All gray value calculations were completed using ImageJ (NIH). For each image, the gray value ratio of laser/healthy was compared with baseline and was reported as a percentage change. The dual normalization, to each image and to the baseline, minimized the technical and the machine biases.

### FA-ICGA

FA-ICGA were obtained after i.v. injection of 1 mL fluorescein 10% (AK Fluor 10% USP, Akorn) and 5 mg ICG (Indocyanine Green 25mg USP, Diagnostic Green) using the Spectralis SD-OCT (Heidelberg Engineering) system. A first-minute movie and 1-, 5-, and 10-minute frames were obtained after the injection.

### AO

#### AO imaging.

AO flood illumination ophthalmoscopy was performed using the rtx-1 machine (Imagine Eyes). Pig positioning was manually adjusted by tilting the cradle in the vertical axis and rotating the rtx-1 in the horizontal plane to capture specific ROI, forming a montage. In total, 5–10 images were collected for each montage; 2-3 montages were collected during imaging for each eye, representing distinct ROIs.

#### AO imaging analysis.

Montages were generated with i2K retina software and were subsequently superimposed with OCT IR fundus images or FA images. While IR images better outlined laser or scaffold locations, FA images allowed for better alignment of the AO images on the retinal and choroidal vasculature. In baseline images, three 80 ***×*** 80 pixel squares were sampled for quantification from a single montage. In laser images, 9 squares of this size were sampled to better represent the variability in cone density in heterogenous-appearing regions. Parameters of density and regularity were averaged for each group of 3 or 9 squares. Images were cropped with Photos (Microsoft) and superimposed with AO montages in Photoshop 2020 (Adobe Systems Inc.). For measurement of PRs, Fiji (NIH) was used to scale images and to calculate cell diameters. Counting of cone PRs was carried out in a semiautomated manner by 3 graders using the AO detect software (AOdetect Mosaic, Imagine Eyes), provided by the company, with custom modifications as follows to improve the accuracy of the algorithm in identifying viable cones in abnormal regions damaged by laser or surgery. Retinal HRF appear as highly reflective, well-circumscribed lesions on OCT images with an equal or greater reflectivity than RPE. These regions are associated with an alteration in choroidal vasculature seen in retinal diseases and pathogenesis. Similarly, we encounter HRF when analyzing AO images of the retina with laser treatment. However, the AO detection software encountered challenges distinguishing HRF from healthy PRs. We manually adjusted the automatic cone detection (AO detect Mosaic, Imagine Eyes) for all the laser-treated retinal images.

### Histology and IHC

At the end of the study, animals were euthanized, and the eyes were collected and processed for histological evaluation. Briefly, 1 eye from each animal was fixed in PFA 4% overnight, cryopreserved, and cut in a cryostat for IHC. In addition, the contralateral eye was fixed in Davidson’s fixative, and the paraffin sections were stained with H&E.

Tissue sections were deparaffinized with xylene (2***×***, 5 minutes) and hydrated by a gradient ethanol series (100 % EtOH [2 min], 90 % EtOH [2 min], 70 % EtOH [2 min], 50 % EtOH [2 min]). Antigen retrieval of Davidson’s solution and formalin-fixed eyes was achieved using a pressure cooker by immersing the sections in 10 mM citrate buffer (pH 6.0) for 12 minutes at 95°C–100°C when buffer containers, each filled with 100 mL of retrieval solution, were preheated for least 10 minutes. Following antigen retrieval, tissue sections were then blocked in PBS containing 10% normal serum (goat or donkey, depending on the secondary), 5% BSA, and 0.5% Triton, and they were incubated overnight at room temperature in primary antibody diluted in blocking buffer (1:100). Primary antibodies against the following proteins were used: IBA1 (catalog 019-19741, WAKO), RPE-65 (catalog ab13826, Abcam) and peanut agglutinin-fluorescein (PNA, 154 Vector Laboratories, FL-1071). Following the overnight primary antibody incubation, the sections were washed 3 times with 1***×*** PBS buffer. Secondary antibodies diluted in blocking buffer (1:500) were added to the sections and incubated in the dark for 1 hour at room temperature. Secondary antibodies used included: Alexa Fluor 555 donkey anti-rabbit (catalog A31572, Thermo Fisher Scientific), Alexa Fluor 555 donkey anti-mouse (catalog A31570, Thermo Fisher Scientific), and Hoechst-33542 (catalog H3570, 1:500, Thermo Fisher Scientific). The sections were washed 3 times with 1***×*** PBS buffer and mounted on a glass slide with Fluoromount-G aqueous mounting medium (catalog 0100-01; Southern Tech) and a glass coverslip. Zeiss Axio Scan.Z1 with a 20***×*** air immersion objective was used to image the entire sections, and ZEISS LSM800 confocal microscope with a 63***×*** oil-immersion objective was used for higher-resolution imaging. H&E-stained slides were used for the manual quantification of nuclei in the outer and inner nuclei layers, RPE, and melanin agglomerates. Nuclei and retinal thickness were evaluated on 5 fields of 100 μm each per eye (*n* = 3 eyes). A portion of the untreated retina within the same slide was used as control and baseline.

### Statistics

Statistical analyses and plot generations were performed in GraphPad (GraphPad Software). Box plots were created to represent the data, with each box extending from minimum to maximum value; the 25th, 75th, and median were represented, and all data points were plotted. All data were first assessed for normality and were, thus, treated as nonnormal. For OCT, OCTA, and AO data, a with mixed-effect model (Restricted Maximum Likelihood [REML]) and Bonferroni multiple-comparison test were used to assess the effect of measurement time point and DC laser on the specified metric. For histology, nuclei quantification, and thickness quantification, 2-way ANOVA and Bonferroni multiple-comparison test were used. *P* < 0.05 was considered significant. *P* values are reported in tables underneath each graph or within the graph. The *P* values within the graphs were reported on top of each graph bar, when different from baseline, with brackets within bars for specific time points and treatments.

### Study approval

All animal procedures were performed in accordance with the guidelines of the Association for Research in Vision and Ophthalmology statement for the use of animals in ophthalmic and vision research, and the procedures received prior approval from the NIH IACUC.

## Author contributions

FB, JA, IB, MF, Rohan Gupta, Rishabh Gupta, and DB developed and tested the laser porcine model. FB, Rohan Gupta, MJP, RJB, AM, DMG, and KB contributed to study design, data analysis, and manuscript writing. KB approved the manuscript.

## Supplementary Material

Supplemental data

Supplemental video 1

## Figures and Tables

**Table 2:**
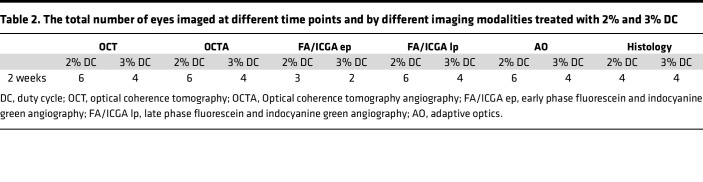
The total number of eyes imaged at different time points and by different imaging modalities treated with 2% and 3% DC

**Table 1:**
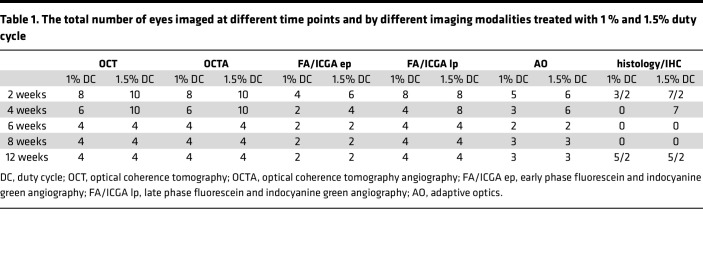
The total number of eyes imaged at different time points and by different imaging modalities treated with 1 % and 1.5% duty cycle

## Version 1 06/08/2023

Electronic publication
