# Supplementary material for: A versatile laser-induced porcine model of outer retinal and choroidal degeneration for preclinical testing
Source: JCI Insight. 2023 Jun 8;8(11):e157654. doi: 10.1172/jci.insight.157654 (PMC10393234; doi:10.1172/jci.insight.157654)
Supplement: Supplemental data [file jciinsight-8-157654-s185.pdf]

**SUPPLEMENTARY FIGURES AND TABLES**

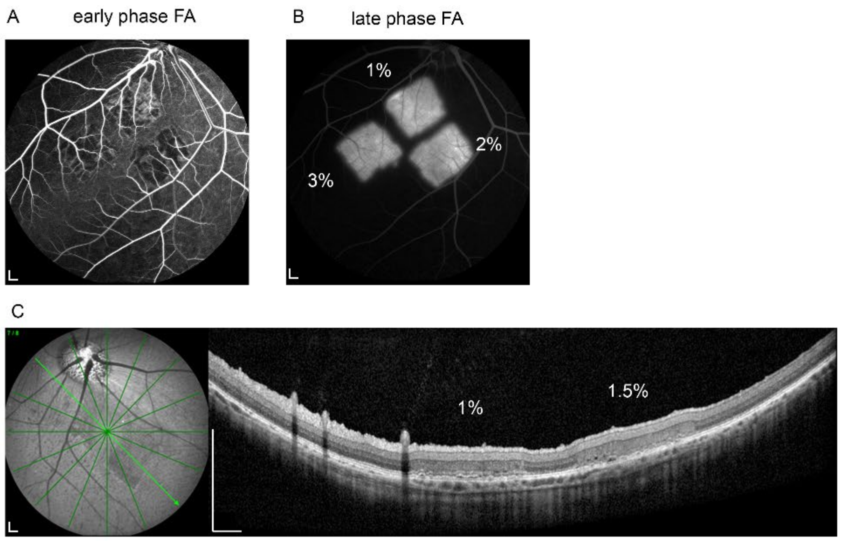


**Supplementary Figure 1.** (A-B) Early (A) and late phase (B) FA evaluated within 3 hours from 1%, 2%, and 3% DC laser treatment. (C) Optical coherent tomography (OCT) scan of the retina imaged within 3 hours from 1% and 1.5% DC laser treatment. FA = fluorescein angiography, DC = duty cycle. Scale bar 1mm.

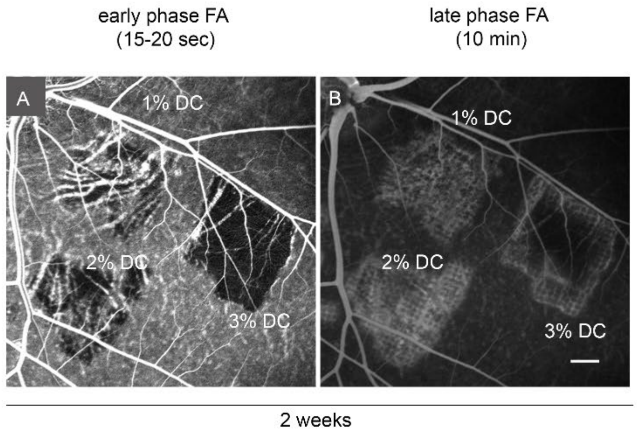


**Supplementary Figure 2.** (A-B) 2 weeks early phase (A) and late phase (B) fluorescein angiography FA images of retina treated in different locations along the visual central streak treated with 1%, 2%, and 3% DC laser power. Scale bars 500 µm.

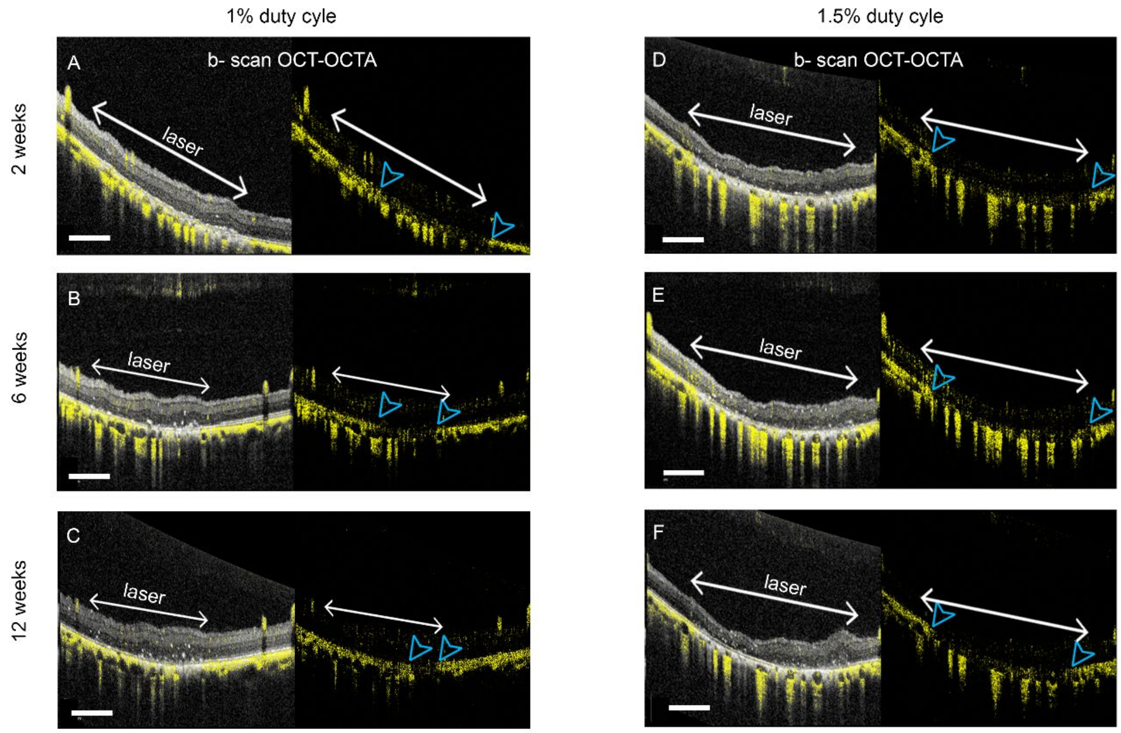


**Supplementary Figure 3**. **B-scan OCT-A images** 2-, 6-, and 12 weeks after 1% (A-C) and 1.5% duty cycle (D-F) laser. White arrows define lasered areas, while blue arrowheads show margins of CC. Scale bars 500 µm.

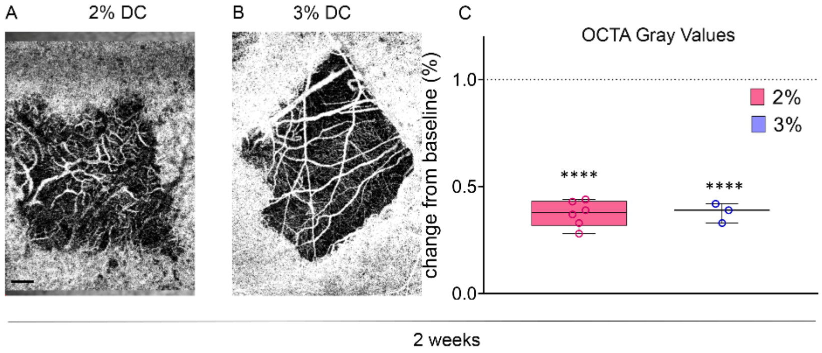


**Supplementary Figure 4.** (A-B) *En face* OCT-A showing choriocapillaris and choroid of 2% (A) and 3% (B) duty cycle laser treated retinas at 2 weeks post treatment. (C) The graph depicts the pixel gray value changes (%) in CC density from baseline to 2 weeks as seen in *en face* images. Box and whiskers represent min-max, 25th and 75th percentile, median and single values. Data were analyzed by two-way ANOVA and Bonferroni multiple comparisons. 3 OCT scans for each eye were analyzed. Number of eyes per condition used for the analysis (C) (n) = 6 (2% DC), 3 (3% DC). *P* values are reported as * $p<0.05$; ** $p<0.005$; *** $p<0.0005$; ***$p<0.0001$. Scale bar 100µm.Scale bars 500 µm.

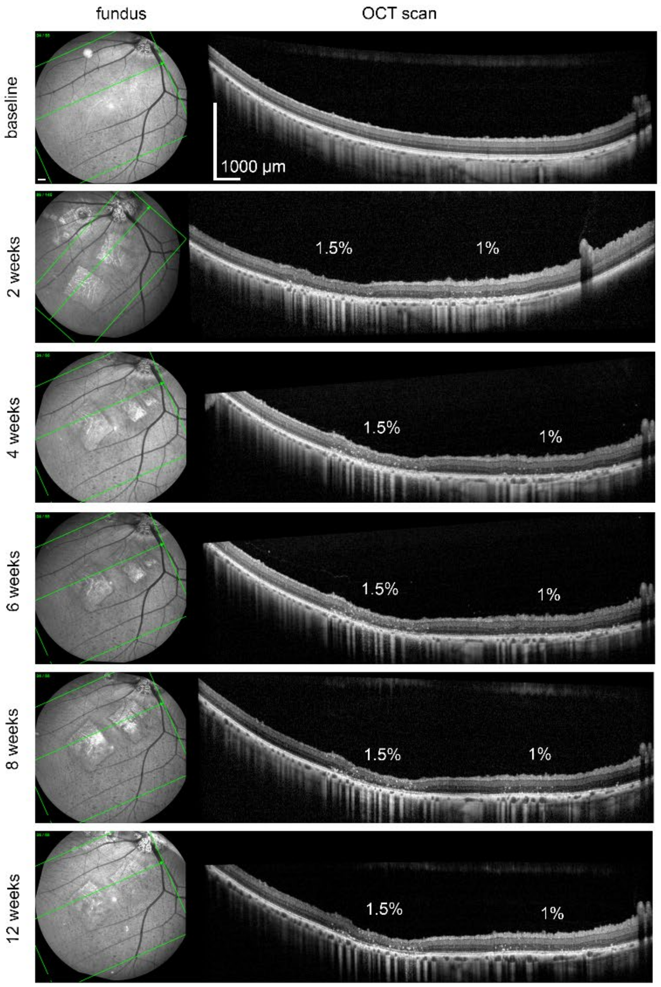


**Supplementary Figure 5**. Representative full OCT images using retina follow up technology of the same retinal region before and 2, 4, 6, 8, and 12 weeks post 1% and 1.5% duty cycle laser treatment. Scale bars 1000 µm

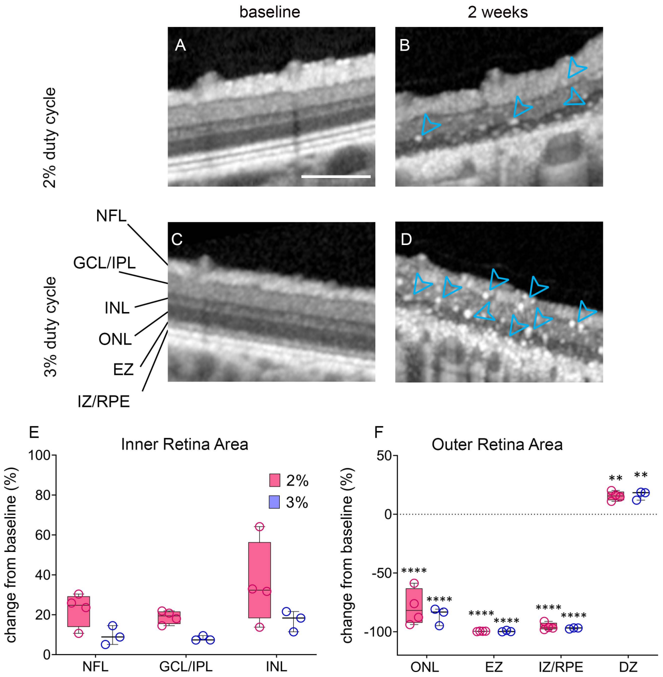


**Supplementary Figure 6.** (A-D) OCT images before (A, C) and 2 weeks after 2% (B) and 3% (D) duty cycle laser treatments. (E-F) Quantification of OCT images from 2% (red) and 3% (blue) duty cycle laser injured retina based on manual segmentation of inner retina (E) and outer retina (F). Percent change in the neuron fiber layer (NFL), ganglion cell layer (GCL)/inner plexiform layer (IPL), inner nuclear layer (INL), outer nuclear layer (ONL), ellipsoid zone (EZ), interdigitation zone (IZ)/retinal pigment epithelium (RPE), and damaged zone (DZ) in 2% and 3% duty cycle laser treated retinas at 2 weeks post laser injury compared to the baseline. Box and whiskers represent min-max, 25th and 75th percentile, median and single values. Data were analyzed by two-way ANOVA and Bonferroni multiple comparisons. 3 OCT scans for each eye were analyzed. Number of eyes per condition used for the analysis (E, F) (n) = 4 (2% DC), 3 (3% DC). *P* values are reported as * $p<0.05$; ** $p<0.005$; *** $p<0.0005$; ***$p<0.0001$. Scale bar 100µm.

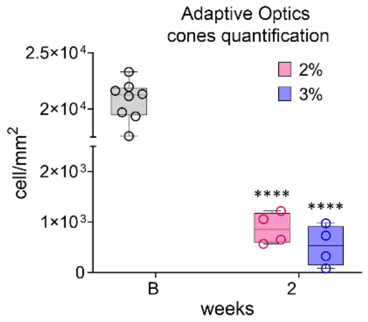


**Supplementary Figure 7.** Adaptive Optics quantification of cone photoreceptors before (baseline, B) and 2 weeks after 2% and 3% DC laser treatment. Box and whiskers represent min-max, 25th and 75th percentile, median and single values. Data were analyzed by two-way ANOVA and Bonferroni multiple comparisons. 5 regions of interest in 3 images for each eye were analyzed. Number of eyes per condition used for the analysis (n) = 4 (2% DC), 3 (3% DC). *P* values are reported as * p<0.05; ** p<0.005; *** p<0.0005; ***p<0.0001.

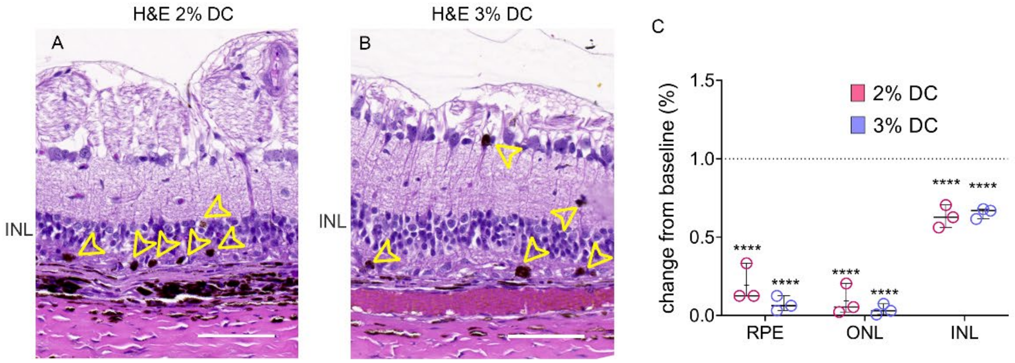


**Supplementary Figure 8**. (A-B) Hematoxylin Eosin (H&E) stained histology of 2% (A) and 3% duty cycle (B) retinas 2 weeks after treatment. (C) Nuclei cell quantification of RPE, ONL, and INL are shown as differences from the baseline. OCT= optical coherence tomography. OCTA= optical coherence tomography angiography. INL= inner nuclear layer. ONL= outer nuclear layer. RPE= retinal pigment epithelium. H&E= Hematoxylin and Eosin staining. Box and whiskers represent min-max, 25th and 75th percentile, median and single values. Data were analyzed by two-way ANOVA and Bonferroni multiple comparisons. 3 slides for each eye were analyzed. Number of eyes per condition used for the analysis (n) = 3. *P* values are reported as * p<0.05; ** p<0.005; *** p<0.0005; ***p<0.0001. Scale bar 50µm.

| animal | eye | laser | date | end of study | fundus |
|---|---|---|---|---|---|
| pig #1 | OD | 1% DC | T0 | 2 weeks | |
| | | 2% DC | T0 | 2 weeks | |
| | | 3% DC | T0 | 2 weeks | |
| | OS | 1% DC | T0 | 2 weeks | |
| | | 2% DC | T0 | 2 weeks | |
| | | 3% DC | T0 | 2 weeks | |
| pig #2 | OD | 1% DC | T0 | 2 weeks | |
| | | 2% DC | T0 | 2 weeks | |
| | | 3% DC | T0 | 2 weeks | |
| | OS | 1% DC | T0 | 2 weeks | |
| | | 2% DC | T0 | 2 weeks | |
| | | 3% DC | T0 | 2 weeks | |
| pig #3 | OD | 1% DC | T0 | 12 weeks | |
| | | 1.5% DC | T0 | 12 weeks | |
| | | 2% DC | T0 | 12 weeks | |
| | OS | 1% DC | T0 | 12 weeks | |
| | | 1.5% DC | T0 | 12 weeks | |
| | | 2% DC | T0 | 12 weeks | |
| pig #4 | OD | 1% DC | T0 | 12 weeks | |
| | | 1.5% DC | T0 | 12 weeks | |
| | OS | 1% DC | T0 | 12 weeks | |
| | | 1.5% DC | T0 | 12 weeks | |
| pig #5 | OD | 1.5% DC | T0 | 4 weeks | |
| | | 1.5% DC | 2 weeks | 4 weeks | |
| | OS | 1.5% DC | T0 | 4 weeks | |
| | | 1.5% DC | 2 weeks | 4 weeks | |
| pig #6 | OD | 1.5% DC | T0 | 4 weeks | |
| | | 1.5% DC | 2 weeks | 4 weeks | |
| | OS | 1.5% DC | T0 | 4 weeks | |
| | | 1.5% DC | 2 weeks | 4 weeks | |
| pig #7 | OD | 1.5% DC | T0 | 4 weeks | |
| | | 1.5% DC | 2 weeks | 4 weeks | |
| | OS | 1.5% DC | T0 | 4 weeks | |
| | | 1.5% DC | 2 weeks | 4 weeks | |

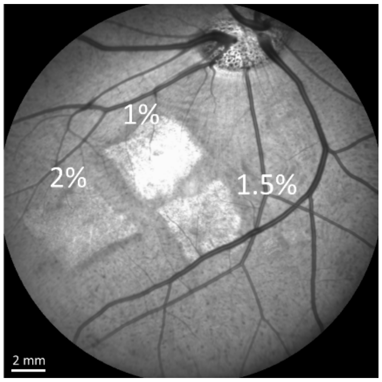


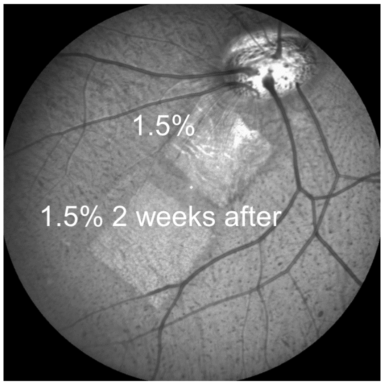


**Supplementary Table 1**. Distribution of laser treatment area for each treated eye. Pigs # 1, 2, 3, and 4 received the laser treatment concurrently (T0). Pigs # 5, 6, and 7 received the second treatment 2 weeks after the first one (T0). Eyes were collected at different endpoints (2, 4, and 12 weeks). Representative images of the eye fundus showing the location of different laser patches are shown. DC = duty cycle

| | Baseline | 1%DC | | | | | 1.5%DC | | | | | 2%DC | 3%DC |
|---|---|---|---|---|---|---|---|---|---|---|---|---|---|
| | | 2 Weeks | 4 Weeks | 6 Weeks | 8 Weeks | 12 Weeks | 2 Weeks | 4 Weeks | 6 Weeks | 8 Weeks | 12 Weeks | 2 Weeks | 2 Weeks |
| Pixel Gray value (0-255) | 170.55 ±51.45 | 106.01 ±26.75 | 112.94 ±44.25 | 134.09 ±25.88 | 165.10 ±36.26 | 158.18 ±51.33 | 84.09 ±24.30 | 91.91 ±30.62 | 77.60 ±14.03 | 89.11 ±32.53 | 91.90 ±31.75 | 74.72 ±14.60 | 83.75 ±14.50 |
| Normalized to control | 0.92 ±0.09 | 0.48 ±0.10 | 0.51 ±0.10 | 0.61 ±0.06 | 0.79 ±0.09 | 0.82 ±0.13 | 0.37 ±0.09 | 0.41 ±0.11 | 0.36 ±0.07 | 0.43 ±0.17 | 0.42 ±0.17 | 0.34 ±0.06 | 0.35 ±0.04 |

**Supplementary table 2:**-Raw pixel gray values and normalized values (average ± standard deviation) of the choriocapillaris and choroid *en face* OCTA quantification before and after 2, 4, 6, 8, and 12 weeks 1% and 1.5% duty cycle and 2 weeks after 2% and 3% DC laser treatment. The control gray value is calculated in an area non affected by laser within the same *en face* image.

| | 1%DC Raw Pixel Values | | | | | | 1.5%DC Raw Pixel Values | | | | | |
|---|---|---|---|---|---|---|---|---|---|---|---|---|
| | Baseline | 2 Weeks | 4 Weeks | 6 Weeks | 8 Weeks | 12 Weeks | Baseline | 2 Weeks | 4 Weeks | 6 Weeks | 8 Weeks | 12 Weeks |
| NFL | 4060.00 | 4695.14 | 4334.25 | 4700.56 | 4480.58 | 4510.67 | 3058.98 | 3441.59 | 4239.53 | 3026.89 | 3540.92 | 3560.17 |
| | ±1925.03 | ±2569.11 | ±1290.99 | ±1165.90 | ±1230.26 | ±1324.75 | ±880.93 | ±1020.92 | ±1491.35 | ±489.77 | ±1140.33 | ±1491.35 |
| GCL/IPL | 4609.67 | 5257.81 | 6171.42 | 6144.56 | 6182.58 | 5897.83 | 6285.63 | 7227.46 | 6868.10 | 6366.44 | 6807.75 | 6612.75 |
| | ±733.44 | ±1101.30 | ±718.29 | ±698.03 | ±876.85 | ±857.32 | ±1341.10 | ±1571.44 | ±1259.77 | ±1037.08 | ±1053.00 | ±1259.77 |
| INL | 2875.42 | 4424.95 | 4891.33 | 4914.67 | 4770.25 | 4986.75 | 3831.00 | 5232.90 | 4776.63 | 4355.89 | 4107.00 | 4088.58 |
| | ±506.74 | ±870.35 | ±515.27 | ±695.32 | ±918.86 | ±901.78 | ±825.06 | ±1342.22 | ±959.26 | ±895.83 | ±1214.02 | ±959.26 |
| ONL | 3338.00 | 1000.19 | 1074.83 | 1067.33 | 1179.67 | 1214.58 | 4087.35 | 977.69 | 880.10 | 586.33 | 754.17 | 989.08 |
| | ±316.64 | ±575.99 | ±595.18 | ±548.84 | ±722.32 | ±926.69 | ±571.56 | ±696.16 | ±668.63 | ±413.55 | ±593.90 | ±668.63 |
| EZ | 1548.00 | 43.95 | 65.33 | 120.33 | 117.17 | 158.92 | 1773.33 | 29.08 | 58.73 | 18.89 | 37.58 | 87.75 |
| | ±238.59 | ±55.31 | ±80.14 | ±150.65 | ±171.11 | ±201.29 | ±359.40 | ±38.94 | ±71.94 | ±22.76 | ±45.93 | ±71.94 |
| IZ/RPE | 1649.46 | 141.19 | 188.33 | 337.44 | 359.08 | 439.33 | 1976.17 | 198.13 | 336.20 | 126.11 | 280.92 | 279.08 |
| | ±268.35 | ±94.78 | ±199.37 | ±192.38 | ±252.93 | ±236.90 | ±414.26 | ±192.18 | ±166.16 | ±77.27 | ±255.87 | ±166.16 |
| DZ | 0.00 | 2725.14 | 2164.58 | 2083.11 | 2042.67 | 1789.25 | 0.00 | 2803.84 | 2369.19 | 2455.00 | 2742.18 | 2430.73 |
| | 0.00 | ±395.35 | ±560.77 | ±437.80 | ±604.62 | ±605.61 | 0.00 | ±784.99 | ±716.74 | ±493.54 | ±519.01 | ±825.35 |
| total | 18080.54 | 17666.10 | 18890.08 | 19368.00 | 19132.00 | 18997.33 | 21012.46 | 19900.77 | 19600.47 | 16935.56 | 18232.00 | 18022.17 |
| | ±448.43 | ±345.42 | ±516.77 | ±446.81 | ±656.85 | ±610.55 | ±680.23 | ±770.52 | ±737.80 | ±475.77 | ±520.80 | ±737.80 |

**Supplementary Table 3**: Raw pixel area values (average ± standard deviation) of each segmented retinal layer before and 2, 4, 6, 8, and 12 weeks 1% and 1.5% duty cycle laser treatment. Neuron fiber layer (NFL), ganglion cell layer (GCL)/inner plexiform layer (IPL), inner nuclear layer (INL), outer nuclear layer (ONL), ellipsoid zone (EZ), interdigitation zone (IZ)/retinal pigment epithelium (RPE), damaged zone (DZ).

| | 1%DC difference from baseline (%) | | | | | 1.5%DC difference from baseline (%) | | | | |
|---|---|---|---|---|---|---|---|---|---|---|
| | 2 Weeks | 4 Weeks | 6 Weeks | 8 Weeks | 12 Weeks | 2 Weeks | 4 Weeks | 6 Weeks | 8 Weeks | 12 Weeks |
| NFL | 13.42 ±10.60 | 18.92 ±15.53 | 20.99 ±13.38 | 23.83 ±18.24 | 23.78 ±18.73 | 19.53 ±20.84 | 23.73 ±13.07 | 24.94 ±22.00 | 29.33 ±22.52 | 28.39 ±29.74 |
| GCL/IPL | 13.33 ±7.78 | 22.04 ±8.54 | 18.99 ±7.47 | 22.41 ±14.78 | 16.48 ±11.08 | 11.04 ±7.35 | 14.80 ±9.60 | 16.72 ±9.91 | 21.84 ±11.86 | 18.01 ±14.78 |
| INL | 57.04 ±19.80 | 55.33 ±22.51 | 47.74 ±22.19 | 49.53 ±22.70 | 57.47 ±27.01 | 35.42 ±15.71 | 28.35 ±21.42 | 28.88 ±29.17 | 19.11 ±32.83 | 20.68 ±35.97 |
| ONL | -70.26 ±17.29 | -68.28 ±17.14 | -67.71 ±15.30 | -65.82 ±19.58 | -64.96 ±26.10 | -75.69 ±17.18 | -78.04 ±14.63 | -83.55 ±10.79 | -79.71 ±14.52 | -73.13 ±16.63 |
| EZ | -97.26 ±3.69 | -96.10 ±4.65 | -93.38 ±7.74 | -93.46 ±8.71 | -90.85 ±10.66 | -98.48 ±2.05 | -96.18 ±6.23 | -98.94 ±1.29 | -97.82 ±2.71 | -94.92 ±4.49 |
| IZ/RPE | -91.24 ±6.23 | -88.82 ±11.06 | -79.32 ±11.60 | -78.41 ±14.23 | -73.21 ±14.24 | -89.35 ±11.45 | -82.27 ±16.44 | -92.16 ±5.60 | -82.87 ±16.03 | -82.93 ±11.00 |
| DZ | 12.06 ±2.17 | 11.44 ±2.61 | 10.80 ±2.33 | 10.63 ±2.72 | 9.58 ±3.60 | 14.01 ±2.78 | 12.47 ±3.95 | 14.40 ±1.88 | 15.37 ±3.00 | 13.52 ±3.58 |

**Supplementary Table 4**: OCT manual segmentation percentage variation (average ± standard deviation) of each retinal layer after 2, 4, 6, 8, and 12 weeks 1% and 1.5% duty cycle compared to baseline. Neuron fiber layer (NFL), ganglion cell layer (GCL)/inner plexiform layer (IPL), inner nuclear layer (INL), outer nuclear layer (ONL), ellipsoid zone (EZ), interdigitation zone (IZ)/retinal pigment epithelium (RPE), damaged zone (DZ).

| | 2%DC Raw Pixel Values | | 3%DC Raw Pixel Values | | 2%DC difference from baseline (%) | 3%DC difference from baseline (%) |
|---|---|---|---|---|---|---|
| | Baseline | 2 Weeks | Baseline | 2 Weeks | 2 Weeks | 2 Weeks |
| NFL | 2947.07 ±1356.89 | 3849.17 ±1517.23 | 2672.56 ±446.10 | 2913.44 ±473.11 | 22.63 ±18.09 | 9.52 ±9.35 |
| GCL/IPL | 5438.87 ±1179.95 | 6178.00 ±1475.71 | 4928.89 ±544.18 | 5325.44 ±613.38 | 18.81 ±7.68 | 8.10 ±5.54 |
| INL | 3310.07 ±699.28 | 4152.83 ±952.99 | 3003.67 ±380.10 | 3491.00 ±367.12 | 33.29 ±29.65 | 17.13 ±12.66 |
| ONL | 3815.80 ±576.09 | 713.83 ±437.61 | 3638.11 ±382.80 | 492.56 ±510.62 | -79.12 ±15.77 | -86.31 ±13.62 |
| EZ | 1833.07 ±331.71 | 21.42 ±33.92 | 1778.11 ±235.22 | 7.67 ±21.17 | -99.62 ±0.53 | -99.58 ±1.16 |
| IZ/RPE | 1895.13 ±392.04 | 135.08 ±98.73 | 1775.89 ±144.16 | 52.11 ±35.97 | -95.65 ±4.10 | -97.04 ±2.04 |
| DZ | 0.00 0.00 | 2779.92 ±706.77 | 0.00 0.00 | 2472.33 ±578.34 | 15.72 ±4.18 | 16.63 ±3.10 |
| total | 19240.00 ±1850.67 | 17830.25 ±1819.80 | 17797.22 ±987.42 | 14754.56 ±1086.32 | | |

**Supplementary Table 5**: Raw pixel area values (average ± standard deviation) and OCT manual segmentation percentage variation (average ± standard deviation) of each segmented retinal layer before and after 2 weeks of 2% and 3% duty cycle laser treatment. Neuron fiber layer (NFL), ganglion cell layer (GCL)/inner plexiform layer (IPL), inner nuclear layer (INL), outer nuclear layer (ONL), ellipsoid zone (EZ), interdigitation zone (IZ)/retinal pigment epithelium (RPE), damaged zone (DZ).
